# The Mechano-Immune-Vascular Activation Loop: An Integrative Conceptual Framework for Positive Feedback in Hypertension

**DOI:** 10.3390/ijms27188048

**Published:** 2026-09-10

**Authors:** Shuang Gao, Beiyan Chen, Xin Chen, Qingping Shi, Mingli Shen, Jieru Han

**Affiliations:** 1 School of Basic Medical Sciences, Heilongjiang University of Chinese Medicine, Harbin 150040, China; gaoshuangacc@163.com (S.G.); chenbeiyanabc@163.com (B.C.); sqp17326705862@163.com (Q.S.); 18309474893@163.com (M.S.); 2 First Clinical Medical College, Heilongjiang University of Chinese Medicine, Harbin 150040, China; radinen@outlook.com

**Keywords:** mechanotransduction, Piezo1, TRPV4, immune activation, vascular inflammation, hypertension, arterial stiffness, positive feedback loop, mechano-immunology, therapeutic targets

## Abstract

Hypertension is increasingly recognized as involving chronic inflammation beyond its hemodynamic origins. This review proposes the Mechano-Immune-Vascular Activation Loop as an integrative conceptual framework—a positive feedback circuit in which aberrant mechanical forces in the hypertensive vascular wall may activate immune cells through mechanosensitive sensors, potentially triggering inflammation that drives vascular stiffening and amplifies mechanical stress. We examine molecular force sensors including piezo-type mechanosensitive ion channel component 1 (Piezo1), transient receptor potential vanilloid 4 (TRPV4), integrins, and Yes-associated protein/transcriptional coactivator with PDZ-binding motif (YAP/TAZ), detailing their expression in vascular cells and immune subsets such as T cells, macrophages, dendritic cells(DCs), and neutrophils. Downstream pathways—calcium-nuclear factor of activated T cells (Ca^2+^-NFAT), nuclear factor kappa-light-chain-enhancer of activated B cells (NF-κB), mitogen-activated protein kinase (MAPK), and Hippo—transduce physical stimuli into inflammatory gene programs. Organ-specific manifestations in the kidney, arteries, heart, and brain are discussed. Therapeutic approaches targeting mechanosensitive channels, repurposed antihypertensives, and mechanical environment modification are evaluated, along with challenges including off-target effects, pathway redundancy, and biomarker needs. By centering mechanotransduction in immune-vascular crosstalk, this framework offers new perspectives on hypertension pathogenesis and points toward interventions that may break the vicious cycle beyond conventional blood pressure reduction.

## 1. Introduction

Hypertension remains the leading modifiable risk factor for cardiovascular morbidity and mortality worldwide, affecting over a billion individuals and contributing to a spectrum of end-organ damage, including heart failure, stroke, and chronic kidney disease [1]. For decades, the pathogenesis of essential hypertension has been understood through the interplay of hemodynamic forces, neurohumoral activation (notably the renin–angiotensin–aldosterone system), and renal sodium handling. However, a growing body of evidence now establishes a third pillar in this framework: low-grade, sterile inflammation [2]. This inflammatory component is not merely a consequence of hypertensive injury but actively participates in disease initiation and progression, driving vascular remodeling and end-organ damage [3].

Concurrently, the vascular wall is a dynamic mechanical environment. Blood pressure elevation exposes endothelial cells, vascular smooth muscle cell layer(VSMCs), and infiltrating immune cells to a complex array of aberrant biophysical forces, including elevated and disturbed shear stress, increased cyclic stretch, and sustained hydrostatic pressure [4]. It is now clear that cells possess a sophisticated molecular machinery to sense these forces—mechanosensitive ion channels like piezo-type mechanosensitive ion channel component 1 (Piezo1)and transient receptor potential vanilloid 4 (TRPV4), adhesion molecules such as integrins, and nuclear transducers like nuclear transducers such as Yes-associated protein/transcriptional coactivator with PDZ-binding motif (YAP/TAZ)—which convert physical stimuli into biochemical signals [5]. This process of mechanotransduction has been shown to regulate endothelial function, vascular smooth muscle cell (VSMC) phenotype, and matrix remodeling.

A critical and emerging concept is that immune cells are themselves adept mechanosensors. T cells, macrophages, dendritic cells (DCs), and neutrophils express Piezo1 and TRPV4, and their functions—including polarization, cytokine production, and cytotoxicity—are directly modulated by mechanical cues like shear stress and tissue stiffness [6,7]. This raises a compelling paradigm: that elevated blood pressure directly triggers immune cell activation via mechanical forces, which in turn exacerbates vascular inflammation and remodeling, creating a self-sustaining pathogenic cycle.

This review integrates these concepts into the “Mechano-Immune-Vascular Activation Loop,” a working model rather than a proven mechanism. We summarize molecular force sensing in vascular and immune cells, delineate mechanical stresses in the hypertensive vascular wall, and detail mechano-immunological profiles of major immune subsets. We then explore downstream pathways—encompassing Ca^2+^/nuclear factor of activated T cells (Ca^2+^-NFAT), nuclear factor kappa-light-chain-enhancer of activated B cells (NF-κB), YAP/TAZ, and mitogen-activated protein kinase (MAPK) cascades—through which force may translate into inflammation. We propose a loop with potential positive feedback, discuss organ-specific manifestations in kidney, arteries, heart, and brain, and evaluate therapeutic implications and remaining challenges for clinical translation.

## 2. Molecular Basis of Mechanical Force Sensing

Mechanical force sensing begins at the plasma membrane, cytoskeleton, and nucleus via conserved machineries [8,9]. In the hypertensive vessel, endothelial cells, smooth muscle cells, and immune cells experience elevated shear, cyclic stretch, and hydrostatic pressure, which are converted into biochemical signals by mechanosensitive ion channels, adhesion molecules, and nuclear transducers [5,10,11]. This section reviews the main components, with emphasis on their expression and function in both vascular and immune cells, and critically assesses their relevance to hypertension.

### 2.1. An Overview of Key Mechanosensory Molecules and Signaling Hubs

Understanding how mechanical forces drive inflammation requires knowing the key molecular sensors and signaling hubs that convert these forces into cellular responses [5,8,9]. This section introduces these components.

#### 2.1.1. Mechanosensitive Ion Channels: The Fast Responders

Ion channels are integral membrane proteins forming pores that allow specific ions (Na^+^, K^+^, Ca^2+^) to cross the cell membrane [12,13]. Mechanosensitive ion channels are directly gated by physical forces—stretch, pressure, or shear stress—making them the body’s most rapid mechanotransducers, converting mechanical stimuli into ionic signals within milliseconds [4,12,14]. Key members include Piezo channels and Transient Receptor Potential (TRP) channels [6,12,14].

Piezo Channels (Piezo1 and piezo-type mechanosensitive ion channel component 2 (Piezo2)): Piezo channels are large trimeric force-gated cation channels; membrane tension directly opens the pore, allowing Ca^2+^ influx [12,14]. It is important to note that Piezo1 is ubiquitously expressed across nearly all tissues and cell types, playing diverse roles from erythrocyte volume regulation to bone formation and baroreception. Piezo2 mediates light touch and proprioception and functions as the primary baroreceptor in nodose ganglia [12,15]. Given the focus of this review on vascular mechano-immunity, we will primarily discuss the roles of Piezo1—along with other key channels—in the context of endothelial cells, vascular smooth muscle cells (VSMCs), and immune cells [2,12,14], as these are the principal cellular players within the hypertensive vascular wall and its associated inflammatory response.

TRP Channels (TRPV4 and transient receptor potential canonical 6 (TRPC6)): TRP channels are non-selective cation channels responding to diverse stimuli, including mechanical force [6]. Similar to Piezo1, TRPV4 is widely distributed; it is activated by stretch, shear stress, and moderate heat, and is prominently expressed in endothelium, VSMCs, and macrophages, where it links to inflammatory signaling [6,16]. TRPC6, activated by membrane stretch, regulates vascular smooth muscle contraction (myogenic tone) and is upregulated in hypertension [17,18].

#### 2.1.2. Adhesion Molecules: Force Transmitters from the ECM to the Cytoskeleton

Integrins are heterodimeric transmembrane receptors linking the extracellular matrix (ECM) to the actin cytoskeleton [4,19,20]. Mechanical force induces integrin clustering and conformational changes, initiating focal adhesion assembly with talin and vinculin [20,21]. Force-dependent conformational changes activate signaling cascades, translating ECM mechanics into signals regulating survival, proliferation, and migration—central to vascular remodeling [4,19,22].

#### 2.1.3. Nuclear Force Transducers and Transcriptional Co-Activators

The Hippo pathway effectors YAP/TAZ are central to mechanosignaling [21,23,24]. LATS1/2 kinases phosphorylate YAP/TAZ, causing cytoplasmic retention [21,23]. Mechanical cues—ECM stiffness, cell spreading, cytoskeletal tension—inhibit this cascade, allowing YAP/TAZ nuclear translocation [21,23,24]. They bind TEAD transcription factors to drive genes for proliferation, survival, and ECM remodeling [23,24], integrating mechanical, hormonal, and metabolic cues [5,21,23].

#### 2.1.4. Master Signaling Hubs: NF-κB and MAPK Pathways

NF-κB, the master inflammatory transcription factor, is held inactive by IκB [25,26]. Mechanical stress activates the IκB kinase (IKK) complex, which phosphorylates IκB for degradation, liberating NF-κB to induce pro-inflammatory cytokines (TNF-α, IL-6), chemokines, and adhesion molecules [25,26,27]. Its activation is a hallmark of chronic inflammation in hypertension [3,26].

MAPKs are serine/threonine kinases regulating proliferation, differentiation, and stress [28,29,30]. The main subfamilies—Extracellular signal-regulated kinase (ERK), c-Jun N-terminal kinase (JNK), and p38—are activated by phosphorylation cascades [28,30,31]. Mechanical stress via integrins or ion channels initiates this cascade [22,28,31], controlling gene expression linked to cell growth and inflammatory cytokine production [28,29,32,33].

### 2.2. Mechanosensitive Ion Channels

Building on this overview, we now detail the specific roles of these ion channels in the context of hypertension. Piezo1 mediates shear-induced nitric oxide (NO) and Ca^2+^-dependent transcriptional programs. In hypertension, Piezo1 is upregulated in podocytes (correlating with injury markers) and is essential for flow-dependent pressure regulation [12]. Critical note: While Piezo1 is clearly required for endothelial function, immune-cell-specific knockout data in hypertensive models are lacking. The use of pharmacological tools—the Piezo1 blocker GsMTx4 and the Piezo1 agonist Yoda1 (a research tool)—is confounded by off-target effects and opposing actions on different cell types. Whether Piezo1 upregulation is protective or pathogenic remains context-dependent.

Piezo2 is the principal baroreceptor sensor in nodose ganglia. Its downregulation via Nedd4-2 in hypertension impairs baroreflex [12]. Critical note: Its role in non-neuronal cells is poorly defined; cardiac upregulation may be compensatory but direct evidence is sparse.

TRPV4—a Ca^2+^-permeable channel—responds to shear and stretch [6]. In macrophages, it triggers the NLR family pyrin domain containing 3 (NLRP3) inflammasome and interleukin (IL)-1β; in neutrophils it promotes neutrophil extracellular trap formation (NETosis) [3].

TRPC6 is a member of the canonical TRP subfamily, forming a tetrameric channel activated by diacylglycerol downstream of phospholipase C signaling. It is permeable to Na^+^ and Ca^2+^ and plays a critical role in regulating myogenic tone and vasoconstriction. In hypertension, TRPC6 is upregulated in resistance arteries, contributing to enhanced vasoconstriction and vascular remodeling [17]. Critical note: Functional redundancy between TRPV4, Piezo1, and TRPC6 is evident, but no study has systematically dissected their relative contributions in vivo. A major limitation is that currently available pharmacological tools (inhibitors/antagonists) lack sufficient selectivity to specifically distinguish among TRPV4, Piezo1, and TRPC6, rendering them unreliable for dissecting the individual functions of these channels in vivo.

Beyond Piezo1, TRPV4, and TRPC6, other ion channels with mechanosensitive properties merit brief mention for their potential contributions to hypertensive inflammation.

First, calcium influx through Piezo1 can activate calcium-dependent potassium channels (KCa), including the small-conductance calcium-activated potassium channel (SKCa),intermediate-conductance calcium-activated potassium channel (IKCa), and large-conductance calcium-activated potassium channel (BKCa). SKCa and IKCa are predominantly expressed in endothelial cells, mediating endothelium-dependent hyperpolarization and vasodilation, while BKCa is highly expressed in vascular smooth muscle cells, regulating membrane potential and myogenic tone. In hypertension, dysregulated KCa function contributes to impaired vasodilation and endothelial dysfunction [34].

Second, the epithelial sodium channel (ENaC) is a heterotrimeric channel (αβγ subunits) belonging to the DEG/ENaC superfamily. It is selectively permeable to Na^+^ and classically defined as a ligand-gated channel. ENaC is most abundant in the renal distal nephron, where it regulates sodium reabsorption and blood pressure; it is also expressed in vascular endothelial cells and immune cells. Importantly, while ENaC is not primarily a mechanosensitive channel, it can exhibit mechanosensitivity in response to shear stress or membrane stretch [35]. Under hypertensive conditions, ENaC activation drives NADPH oxidase-dependent formation of isolevuglandins (IsoLGs), triggering NLRP3 inflammasome activation and T-cell polarization [36]. Detailed mechanistic dissection of these channels warrants future investigation.

### 2.3. Mechanosensitive Adhesion Molecules and Cytoskeleton

Integrins (αvβ3, α5β1) connect ECM to actin, activate focal adhesion kinase (FAK) and downstream MAPK/PI3K, and regulate immune cell migration and VSMC phenotype switching. Talin and vinculin undergo force-dependent conformational changes [20]. Critical note: Integrin-mediated mechanotransduction is often studied using rigid substrates that do not replicate the dynamic stiffness of hypertensive vessels [37]. The relative contribution of outside-in versus inside-out signaling in hypertension is unresolved.

### 2.4. Nuclear Force Sensing

The linker of nucleoskeleton and cytoskeleton (LINC) complex transmits force to the nucleus, affecting chromatin and YAP/TAZ activity. YAP/TAZ are regulated by ECM stiffness and stretch; they promote T helper 17 cell (Th17) differentiation, M1 polarization, and endothelial inflammation [21,38,39]. Critical note: YAP/TAZ are promiscuous integrators of multiple signals (mechanical, hormonal, metabolic). Disentangling the specific mechanical input from other stimuli (e.g., angiotensin II (Ang II), salt) is a major challenge. Most data come from cancer or developmental biology and may not translate to hypertensive immunity.

These molecular sensors can be conceptually grouped into three functional categories based on their primary site of action and mode of force transduction. First, mechanosensitive ion channels (Piezo1, Piezo2, TRPV4, TRPC6, among others) are directly gated by membrane tension or stretch, providing the most rapid Ca^2+^-dependent responses to acute mechanical perturbations. Second, adhesion molecules such as integrins, talin, and vinculin connect the extracellular matrix to the actin cytoskeleton [40], transmitting force across the plasma membrane and activating downstream kinase cascades (e.g., FAK–MAPK) that regulate cell migration, proliferation, and phenotype switching. Third, nuclear force transducers, including the LINC complex and the transcriptional co-activators YAP/TAZ, relay mechanical signals from the cytoskeleton into the nucleus, where they reprogram gene expression over longer time scales.

## 3. Mechanical Forces in the Hypertensive Vascular Wall

Hypertensive vessels experience four primary mechanical stresses—shear stress (frictional force from blood flow, elevated and disturbed in hypertension), cyclic stretch (pulsatile deformation, increased amplitude and frequency), hydrostatic pressure (luminal pressure, uniformly raised), and interstitial fluid pressure (elevated in tissues like the kidney)—each acting predominantly on endothelial cells, VSMCs/adventitia, all layers, or tissue-resident cells, respectively; shear stress normally maintains endothelial homeostasis via Piezo1-mediated NO (10–70 dyn/cm^2^), but in hypertension disturbed/oscillatory flow at bifurcations upregulates adhesion molecules and promotes leukocyte adhesion [3,14,41,42,43], while cyclic stretch (up to 15 strain) activates Piezo1/TRPV4/integrins in VSMCs, driving phenotypic switching [22,44], and also triggers podocyte injury via Ras-related C3 botulinum toxin substrate 1 (Rac1) [45,46]; hydrostatic pressure is most damaging in renal microcirculation and portal hypertension [47], and interstitial pressure activates resident macrophages via NLRP3 [48].

These forces are not uniform along the vascular tree: large elastic arteries (aorta, carotids) endure the highest cyclic stretch and oscillatory shear, favoring endothelial dysfunction and stiffening [49]; resistance arteries exhibit enhanced myogenic tone and TRPC6-mediated vasoconstriction; and the renal microcirculation, with glomerular pressure ~45–60 mmHg, upregulates Piezo1 in podocytes, driving albuminuria [45,46]—while circulating leukocytes sense luminal shear, extravasated cells encounter wall stiffness and interstitial pressure, and tissue-resident macrophages directly sense matrix deformation, creating segment-specific inflammatory niches.

Superimposed are critical spatiotemporal dimensions: acute mechanical stress triggers protective NO-mediated vasodilation and transient immune responses, whereas chronic overload (months to years) induces maladaptive VSMC remodeling, ECM deposition, and persistent immune infiltration, establishing a self-perpetuating loop [22,49,50]. Laminar shear is atheroprotective, but oscillatory/turbulent flow promotes endothelial activation and monocyte adhesion [41,42,43]; and increased stretch frequency (tachycardia) may enhance mechanosensitive kinase activation and shift the NO/superoxide balance toward oxidative stress [51,52]. Critically, however, the quantitative thresholds for “pathological” forces remain undefined for each cell type and vascular bed—values like 100 dyn/cm^2^ or 20% strain are derived from computational estimates or healthy physiology, not hypertensive tissues [53]. The causal link between regional mechanical heterogeneity and local immune infiltration is largely correlative, with no segment-specific mechanical manipulation to prove direct causation [54]; the acute-protective versus chronic-pathological distinction is inferred from temporal extrapolation, yet most in vitro models apply sudden step-changes rather than the progressive overload of human hypertension; frequency-dependent effects are supported by limited computational or animal data, and whether heart rate reduction per se (independent of BP lowering) attenuates mechano-immune activation remains untested; finally, the integration of regional, temporal and frequency variables is completely unexplored, so while the conceptual framework is attractive, therapeutic strategies aimed at normalizing these mechanical signals (BP control, rate reduction, or local modulation) currently rest on phenomenological correlations rather than rigorous causal evidence (Table 1).

## 4. Immune Cells as Mechanical Force Sensors

Immune cells are active mechanosensors that directly respond to the abnormal shear stress, cyclic stretch and hydrostatic pressure of hypertension through dedicated molecular machinery [38,55,56,57], with each major subset exhibiting distinct mechano-immunological profiles: T cells express Piezo1, which mediates shear-induced Ca^2+^ influx that synergizes with T cell receptor (TCR) signaling to lower activation thresholds and drives calcineurin-NFAT-dependent interferon-gamma (IFN-γ)/IL-17 production [58,59], promoting Th17 skewing under high mechanical load while low forces favor regulatory T cells (Treg) maintenance [60]; macrophages utilize TRPV4 and Piezo1 to translate stretch into Ca^2+^-dependent NLRP3 inflammasome activation and IL-1β release [61,62], and TRPV4-NF-κB signaling promotes M1 polarization, whereas lower mechanical cues favor reparative M2 phenotypes [63], with mechanical stress also enhancing oxidized LDL uptake and foam cell formation [6,12]; DCs employ Piezo1 to boost maturation, antigen cross-presentation and IL-6 secretion, thereby potentiating Th17 differentiation [64]; neutrophils activate Piezo1 and TRPV4 during microvascular deformation, triggering calpain-mediated cytoskeletal rearrangement and NETosis, which directly injures endothelium [56]; B cells express Piezo1 but their role in hypertensive mechano-immunology remains virtually unexplored; and tissue-resident immune cells—embedded in distinct mechanical niches such as the stiff aortic wall, high-pressure renal interstitium or cyclically stretched myocardium [65]—experience mechanical cues fundamentally different from those of circulating leukocytes, likely imprinting organ-specific functional programs.

**Figure 1:**
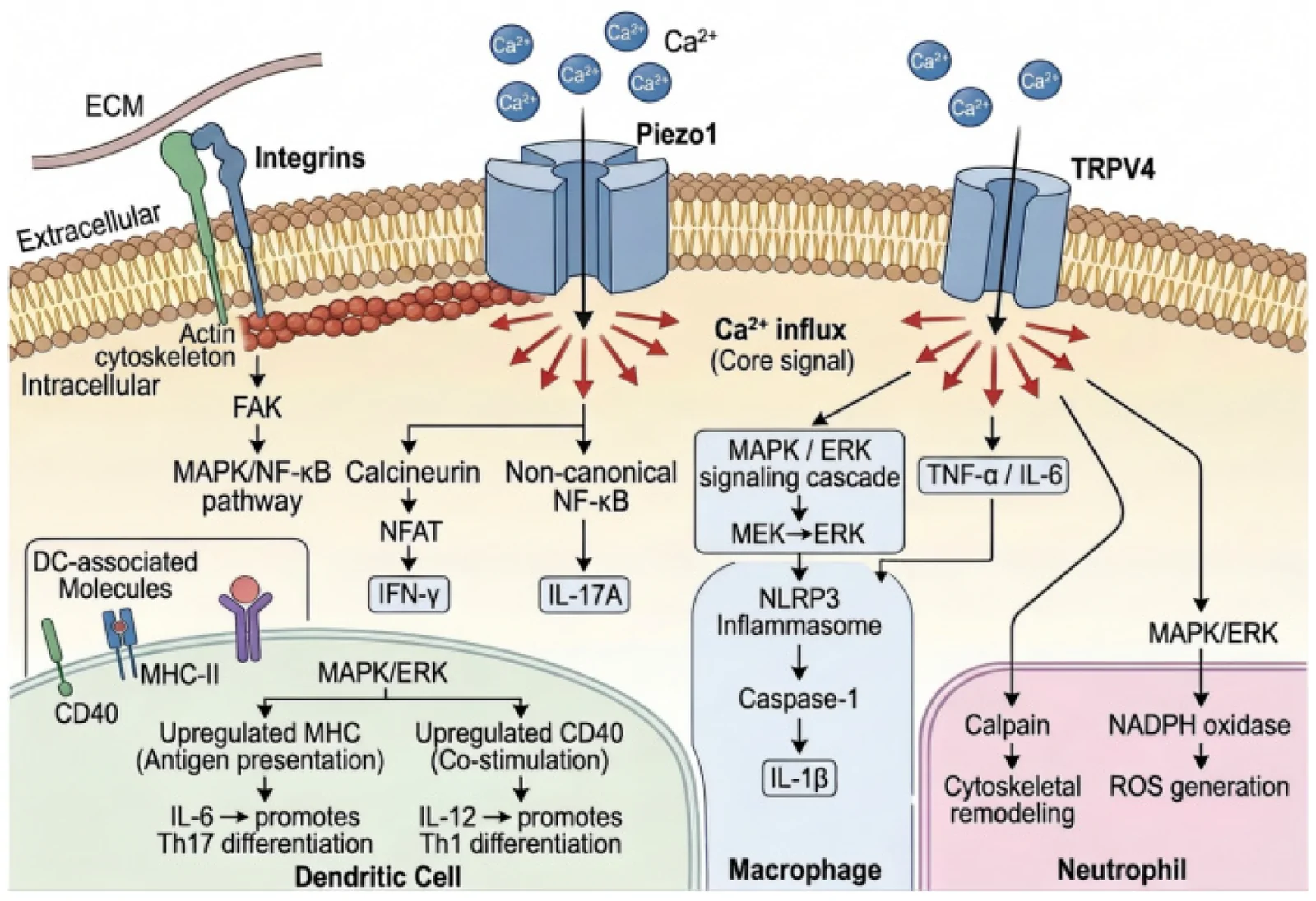
Mechanosensitive molecules and downstream signaling pathways in major immune cell subsets under hypertensive mechanical stress. This figure depicts the mechanosensitive non-selective cation channels (Piezo1, TRPV4), adhesion molecules (integrins), and signaling cascades activated in key immune cell types. T cells express Piezo1, which triggers a Ca^2+^-dependent calcineurin-NFAT pathway, promoting Th17 differentiation and production of IFN-γ and IL-17. Macrophages utilize Piezo1 and TRPV4 to activate the NLRP3 inflammasome and NF-κB signaling, leading to M1 polarization and IL-1β release. Dendritic cells (DCs) use Piezo1 to enhance maturation and IL-6 secretion, which potentiates Th17 responses. Neutrophils activate Piezo1 and TRPV4, leading to calpain-mediated NETosis and endothelial injury. The figure highlights the cell-type-specific responses that link mechanical sensing to distinct pro-inflammatory effector functions. Figure 1 was created with Figdraw (www.figdraw.com). Abbreviations: ECM, extracellular matrix; Piezo1, piezo-type mechanosensitive ion channel component 1; TRPV4, transient receptor potential vanilloid 4; FAK, focal adhesion kinase; MAPK, mitogen-activated protein kinase; NF-κB, nuclear factor kappa-light-chain-enhancer of activated B cells; NFAT, nuclear factor of activated T cells; IFN-γ, interferon-gamma; IL, interleukin; ERK, extracellular signal-regulated kinase; MEK, mitogen-activated protein kinase kinase; TNF-α, tumor necrosis factor-alpha; DC, dendritic cell; MHC, major histocompatibility complex; CD40, cluster of differentiation 40; Th17, T helper 17 cell; NLRP3, NLR family pyrin domain containing 3; NADPH, nicotinamide adenine dinucleotide phosphate hydrogen; ROS, reactive oxygen species.

Critically, however, the vast majority of these findings derive from in vitro systems (fluid-shear chambers, stretch devices, pharmacological agonists like Yoda1) that fail to replicate the chronic, complex, and multidimensional mechanical environment of hypertension in vivo; immune-cell-specific conditional knockouts of Piezo1 or TRPV4 have not been tested in hypertensive models, leaving the causal contribution of each cell type unproven; the reported M1/M2 and Th17/Treg shifts are largely inferred from correlative or indirect data [60,63] and direct evidence from human hypertensive patients—such as mechanosensor expression on circulating immune cells or real-time tracking of mechanical signal transduction—is almost entirely absent; moreover, the functional redundancy among Piezo1, TRPV4, and integrins raises doubts about whether targeting any single channel can effectively disrupt the loop, and the interpretability of inhibitor studies is confounded by off-target effects and opposing cell-type-specific actions. Thus, while the concept that immune cells are mechanically programmed is compelling, the field currently rests more on plausible in vitro phenomenology than on definitive in vivo causal proof [56,61,66], and major gaps remain regarding cell-type specificity, mechanistic hierarchy, and translational relevance to human hypertension (Figure 1 and Table 2) [67].

## 5. Mechanical Force-Driven Signaling in Immune Activation

Mechanical forces do not simply trigger a single linear pathway; they engage a complex, interconnected signaling network that converges on key transcription factors and effector molecules. At the heart of this network is Ca^2+^—the prototypical second messenger of mechanotransduction—alongside NF-κB, YAP/TAZ, and MAPK cascades. These pathways do not operate in isolation; they intersect with classical immune signals (TCR, toll-like receptor (TLR), cytokine receptors) to lower activation thresholds, amplify inflammatory outputs, and establish a self-reinforcing mechano-immune loop.

### 5.1. Ca^2+^ Signaling—The Core Second Messenger of Mechano-Immunology

Calcium ions serve as the most immediate and universal second messengers in mechano-immunology, as Piezo1 and TRPV4 permit rapid extracellular Ca^2+^ influx [62,68] that branches into four major downstream effector axes: the calcineurin-NFAT pathway, which dephosphorylates NFAT and synergizes with AP-1 to drive transcription of IL-2, IFN-γ and IL-17A in T cells, with Piezo1-mediated Ca^2+^ entry lowering the TCR activation threshold such that suboptimal antigen engagement becomes sufficient for full transcriptional responses [3,12]; the Ca^2+^/calmodulin-dependent protein kinase (CaMK)-cAMP response element-binding protein (CREB) axis, which promotes cell survival, proliferation, and metabolic adaptation through genes involved in mitochondrial biogenesis and nutrient uptake, supporting the energetic demands of sustained immune activation in the chronically inflamed hypertensive environment [3]; the NLRP3 inflammasome–caspase-1–IL-1β pathway, where Ca^2+^-induced mitochondrial dysfunction and reactive oxygen species (ROS) production facilitate NLRP3 oligomerization, with TRPV4 specifically linked to this process in macrophages (and Piezo1 contributing in a cell-type-specific manner), making this a central mechanism for sterile inflammation where damage-associated molecular patterns (DAMPs) and mechanical stress converge [6]; and the cytosolic phospholipase A2 (cPLA2)-arachidonic acid cascade, which liberates arachidonic acid from membrane phospholipids for cyclooxygenase/lipoxygenase conversion into prostaglandins and leukotrienes that promote vascular permeability, leukocyte recruitment, and pain signaling in chronic hypertension [6,72].

Critically, however, this canonical branching model, while mechanistically attractive, suffers from several major limitations: the linear diagram oversimplifies extensive cross-talk between pathways (e.g., NFAT and NF-κB cooperate, CaMK can modulate NLRP3, and arachidonic acid metabolites feedback on Ca^2+^ channels); the concept that Ca^2+^ “lowers the TCR threshold” is derived almost exclusively from in vitro fluid-shear experiments using Jurkat cells or artificial substrates [56,61,73], with no in vivo proof that circulating T cells experience sufficient shear to achieve this effect; the relative contribution of Piezo1 versus TRPV4 versus other Ca^2+^-permeable channels (e.g., calcium release-activated calcium channel protein 1, P2X purinoceptor 7 (P2X7)) to the overall Ca^2+^ signal in each immune cell subset under hypertensive conditions is completely unknown, and pharmacological dissection is confounded by the non-selectivity of available inhibitors; the NLRP3–IL-1β axis is convincingly linked to mechanical stress in macrophages [61], but whether Piezo1 or TRPV4 dominates in vivo remains unresolved, and most data come from acute lipopolysaccharide-primed models that do not replicate the chronic [61,74], low-grade sterile inflammation of hypertension; the CaMK–CREB and cPLA2 pathways are largely inferred from non-immune or non-hypertensive systems (neuroscience, cancer, or acute inflammation), and their specific relevance to hypertensive immune activation is essentially unvalidated; and finally, the field has not established whether the Ca^2+^ signature—amplitude, frequency, duration—differs between physiological (homeostatic) and pathological (hypertensive) mechanical forces, which is a fundamental gap because the same Ca^2+^ rise can trigger NO-mediated vasodilation in endothelium versus IL-17 production in T cells [75,76], depending on cell type and context.

Thus, while Ca^2+^ unquestionably acts as a central branching node integrating mechanical input into inflammatory outputs, the current understanding remains largely descriptive and pathway-centric, with major unanswered questions regarding in vivo causality, pathway hierarchy, cell-type specificity, and the qualitative nature of the Ca^2+^ signal itself that must be addressed.

In vitro, cyclic stretch activates Piezo1 in podocytes (Rac1/PAI-1/SGK1) [45] and drives VSMC phenotypic switching [22,44]. Yoda1-induced Piezo1 agonism triggers Ca^2+^ influx, ZAP70 phosphorylation, and lowers TCR threshold in T cells [68,69], while in macrophages it activates NLRP3–IL-1β. However, these findings derive from reductionist systems that fail to replicate chronic hypertensive mechanics.

### 5.2. NF-κB Pathway Activation by Mechanical Force

NF-κB is a master inflammatory regulator activated by mechanical stress through three convergent routes. IKK axis: ECM stiffness or stretch engages integrins, activating FAK, which recruits IKK to phosphorylate IκBα, leading to its degradation and NF-κB nuclear translocation [39]. This pathway drives IL-6, TNF-α, intercellular adhesion molecule-1 (ICAM-1) and vascular cell adhesion molecule-1 (VCAM-1) expression in VSMCs and macrophages within stiffened hypertensive vessels [19,22,25]. ROS-dependent pathway: mechanical stretch and shear generate ROS via nicotinamide adenine dinucleotide phosphate hydrogen (NADPH)oxidases (Nox1/2/4) and mitochondrial dysfunction, which activate IKK by oxidizing upstream kinases or inhibiting phosphatases; in hypertensive mice, vascular oxidative stress produces isoketal adducts that activate DCs and T cells, fueling NF-κB-driven inflammation [26]. Piezo1–Ca^2+^–protein kinase C (PKC) (non-canonical): in immune cells, Piezo1-mediated Ca^2+^ influx activates PKC isoforms, which phosphorylate and activate IKK independently of integrin-FAK, providing a rapid pathway that allows circulating immune cells to respond to shear stress before extravasation [77]. Collectively, these mechanisms ensure NF-κB activation by virtually all forms of hypertensive mechanical stress—shear, stretch, stiffness, and pressure—making it a central transcriptional hub for mechano-induced inflammation.

While the convergence of multiple pathways on NF-κB seems robust, this redundancy raises a critical therapeutic challenge—blocking a single upstream route (e.g., integrin-FAK or Piezo1) is unlikely to fully suppress NF-κB activation, as others may compensate. Moreover, the relative contribution of each pathway in vivo remains unknown; most evidence for the Piezo1–PKC route comes from in vitro immune cell studies, and whether this non-canonical pathway operates under chronic hypertensive conditions has never been directly tested. The ROS–NF-κB link is well established but is confounded by the fact that ROS also activate other pro-inflammatory pathways (e.g., NLRP3, MAPK), making it difficult to attribute NF-κB effects specifically to oxidative stress. Finally, all three pathways converge on IKK, yet we lack tools to distinguish IKK activation driven by mechanical versus biochemical (e.g., TNF-α, IL-1β) stimuli in vivo, so its activation in hypertension is not definitive proof of a mechano-specific effect.

Parallel-plate flow chamber studies show that disturbed shear upregulates endothelial ICAM-1/VCAM-1, promoting monocyte adhesion and transmigration [27,41]. This establishes a direct in vitro link between hemodynamic forces and immune activation.

### 5.3. YAP/TAZ—Mechanically Regulated Transcriptional Co-Activators

YAP/TAZ are Hippo pathway effectors whose activity is exquisitely sensitive to sustained mechanical cues, integrating them into longer-term transcriptional reprogramming. Under low mechanical load, Large Tumor Suppressor Kinase 1/2 (LATS1/2) phosphorylate YAP/TAZ, promoting cytoplasmic retention and degradation; high mechanical forces—stiff ECM, cyclic stretch, or hydrostatic pressure—inhibit LATS1/2, allowing YAP/TAZ dephosphorylation, nuclear translocation, and TEA domain transcription factor -dependent transcription of genes regulating proliferation, ECM remodeling, and inflammation [23,47]. In immune cells, YAP/TAZ promote M1-like pro-inflammatory gene expression (IL-1β, iNOS, tumor necrosis factor-alpha (TNF-α)) in macrophages while suppressing M2 markers [78,79]; in T cells, they drive Th17 differentiation (e.g., in salt-sensitive hypertension models) and may influence Treg function, though mechanisms remain unclear [80,81]; in DCs, their roles are even less characterized. YAP/TAZ also physically interact with NF-κB p65 to synergistically amplify IL-6, IL-8 and monocyte chemoattractant protein-1 (MCP-1) expression [74], and angiotensin II activates YAP/TAZ via protein phosphatase 2A (PP2A)-dependent dephosphorylation in endothelium, promoting vascular inflammation [24].

YAP/TAZ are notoriously promiscuous integrators of mechanical, hormonal, and metabolic signals—disentangling the specific mechanical input from Ang II, salt, or inflammatory cytokines in vivo is a formidable challenge that has not been adequately addressed. Most immune cell data come from cancer or developmental biology models [82,83], with only sparse validation in hypertensive contexts; the macrophage M1/M2 effect is largely inferred from in vitro stiffness or stretch experiments [57,61], and whether YAP/TAZ nuclear translocation actually drives macrophage polarization in the hypertensive vessel wall remains unproven. The NF-κB synergy is mechanistically intriguing but has been demonstrated primarily in endothelial or cancer cell lines, not in immune cells under hypertensive conditions [84]. Moreover, we lack immune-cell-specific YAP/TAZ knockout studies in hypertensive models, so their causal contribution to T cell skewing or macrophage activation is correlative at best. Finally, the reported Th17 skewing by high salt is difficult to attribute specifically to mechanical stress versus osmotic effects, and whether YAP/TAZ act directly on retinoic acid receptor-related orphan receptor gamma t or via IL-6/ signal transducer and activator of transcription 3 (STAT3) is unresolved—a distinction that matters for therapeutic targeting.

### 5.4. MAPK Pathway Activation by Mechanical Force

MAPKs are activated by mechanical stress through both integrin-dependent and integrin-independent routes [28]. These kinases regulate immediate-early gene expression, cell proliferation, and inflammatory mediator production.

Integrin → FAK → Ras → Raf → mitogen-activated protein kinase kinase (MEK) → ERK. As with NF-κB, integrin-FAK signaling initiates the classical Ras-Raf-MEK-ERK cascade. ERK phosphorylates transcription factors such as Elk-1 and c-Fos, which participate in AP-1 complex formation. In VSMCs, ERK activation drives proliferation and synthetic phenotype switching [29]; in macrophages, it enhances cytokine production.

Mechanical force → JNK/p38 → AP-1. JNK and p38 are stress-activated MAPKs that respond to mechanical stretch, shear stress, and osmotic pressure. Their activation can occur via upstream kinases (e.g., MAP kinase kinase (MKK)4/7 for JNK, MKK3/6 for p38) that are triggered by ROS, Ca^2+^ signals, or integrin clustering [31]. Once activated, JNK and p38 phosphorylate c-Jun and activating transcription factor 2, respectively, both components of AP-1 [32]. AP-1 cooperates with NF-AT and NF-κB to drive a broad inflammatory transcriptome [33].

Rho family GTPases as upstream mediators. Mechanical forces also activate small GTPases such as Ras homolog family member A (RhoA), Rac1, and Cdc42, which in turn regulate MAPK pathways. For example, cyclic stretch activates Rac1 in podocytes, leading to activation of p38 and JNK and upregulation of injury markers PAI-1 and SGK1 [45]. This pathway is directly relevant to hypertensive nephropathy, where mechanical stretch of podocytes drives local inflammation and glomerular injury.

### 5.5. Crosstalk Between Mechanical Signals and Classical Immune Signaling Pathways

Mechanical signals do not act in isolation; they potentiate classical immune pathways through three levels of crosstalk. First, fluid shear stress lowers the TCR activation threshold via Piezo1–Ca^2+^–calcineurin–NFAT, enhancing ZAP70 phosphorylation and cytokine production such that suboptimal antigen engagement becomes sufficient to activate T cells—potentially priming circulating T cells to respond to weak or self-antigens in hypertensive patients. Second, mechanical stress synergizes with TLR signaling by upregulating TLR2/4 expression on macrophages and endothelium, generating endogenous DAMPs (Hsp60, fibronectin fragments, ATP) that engage TLRs [85,86], and potentiating TLR-induced NF-κB and interferon regulatory factor activation through Piezo1/TRPV4 Ca^2+^ signals; in DCs, combined mechanical and TLR4 stimulation synergistically increases major histocompatibility complex (MHC) and costimulatory molecule expression, enhancing antigen presentation [73,83]. Third, mechanical signals intersect with cytokine receptor pathways: cyclic stretch enhances IL-6–induced STAT3 phosphorylation in VSMCs [87], shear stress modulates IL-4 receptor expression on macrophages, and mechanical forces influence cytokine receptor expression/shedding, altering immune cell sensitivity to paracrine signals [57]. Ultimately, all these pathways converge on shared transcription factors—NFAT, NF-κB, activator protein-1 (AP-1), and YAP/TAZ—which integrate multiple inputs, producing synergistic (not merely additive) inflammatory gene programs that drive vascular remodeling and hypertension progression [1,24].

Critical note: While this crosstalk model is conceptually powerful, its experimental foundation is uneven. The TCR–Piezo1 synergy rests almost exclusively on in vitro fluid-shear experiments using Jurkat cells or artificial substrates [82]; no in vivo evidence shows circulating T cells experience sufficient shear to lower TCR thresholds in hypertensive patients. The TLR–mechanical synergy has stronger support [26], but mechanical versus biochemical TLR ligands are inseparable in vivo. Cytokine receptor crosstalk remains the weakest link, largely inferred from acute stretch cultures. Crucially, all pathways converge on shared transcription factors also activated by Ang II, TNF-α, or IL-17, so their activation does not prove mechano-specific synergy—a concept poorly quantified without systematic dose–response studies. The therapeutic implication of pathway redundancy remains untested.

**Figure 2:**
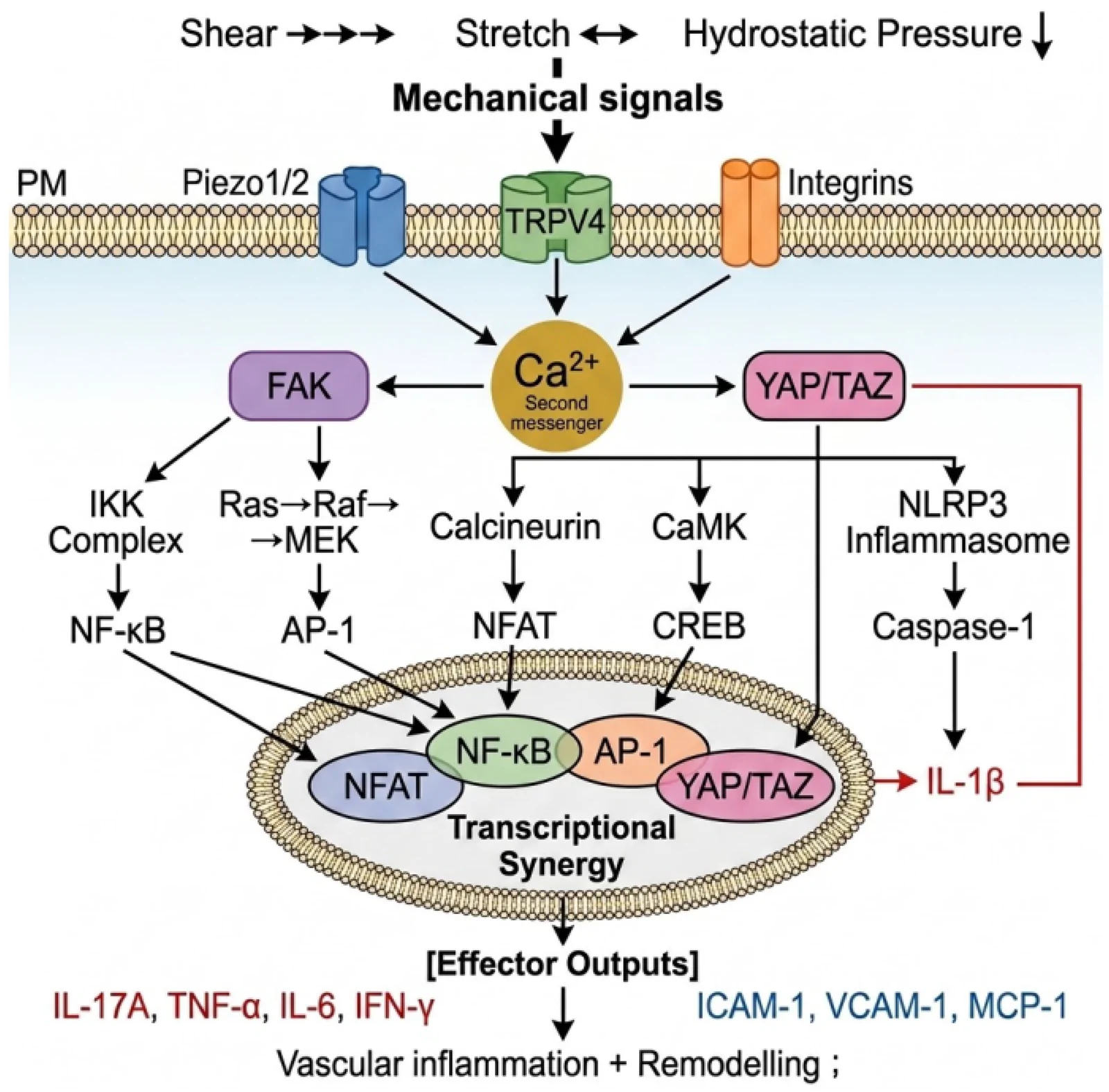
Integrated signaling pathways from mechanical force to immune activation. This schematic illustrates how biomechanical stimuli—including shear stress, cyclic stretch, and hydrostatic pressure—are transduced into intracellular signaling cascades that drive pro-inflammatory gene expression in vascular and immune cells. Figure 2 was created with Figdraw (www.figdraw.com). Abbreviations: Piezo1/2, piezo-type mechanosensitive ion channel component 1/2; TRPV4, transient receptor potential vanilloid 4; FAK, focal adhesion kinase; YAP/TAZ, Yes-associated protein/transcriptional coactivator with PDZ-binding motif; IKK, IκB kinase; NF-κB, nuclear factor kappa-light-chain-enhancer of activated B cells; Ras, Rat sarcoma virus protein; Raf, Rapidly Accelerated Fibrosarcoma kinase; MEK, mitogen-activated protein kinase kinase; AP-1, activator protein-1; NFAT, nuclear factor of activated T cells; CaMK, Ca2+/calmodulin-dependent protein kinase; CREB, cAMP response element-binding protei; NLRP3, NLR family pyrin domain containing 3; IL, interleukin; TNF-α, tumor necrosis factor-alpha; IFN-γ, interferon-gamma; ICAM-1, intercellular adhesion molecule-1; VCAM-1, vascular cell adhesion molecule-1; MCP-1, monocyte chemoattractant protein-1.

In DCs, combined Piezo1 mechanical stimulation and TLR4 ligation synergistically increase MHC class I, cluster of differentiation 40(CD40) and IL-6, enhancing antigen cross-presentation [70]. This has been demonstrated in vitro, but its in vivo relevance to hypertensive DCs remains unvalidated (Figure 2).

## 6. The Mechano-Immune Activation Loop in Hypertension

The preceding sections have established that hypertension generates abnormal mechanical forces, that these forces are sensed by dedicated molecular machinery in both vascular and immune cells, and that they trigger signaling cascades driving inflammatory gene expression [88]. Here, we synthesize these elements into an integrative conceptual framework: the mechano-immune activation loop. We propose this as a working model—a potential positive, self-sustaining circuit in which elevated blood pressure produces pathological mechanical stress, which may activate immune cells; the resulting inflammation could drive vascular remodeling and stiffening; and the altered vascular mechanics may further elevate blood pressure, thereby closing the loop. It is important to emphasize that this model, while conceptually compelling, is supported predominantly by preclinical and in vitro data, with limited direct causal evidence from human hypertension. This section delineates the proposed loop architecture, identifies its critical molecular nodes, characterizes its putative positive feedback properties, and examines its potential interactions with other well-established hypertensive pathways.

It is important to acknowledge that the evidence supporting this proposed framework derives from distinct experimental and clinical contexts, with varying degrees of direct relevance to human systemic arterial hypertension. First, direct evidence from systemic arterial hypertension models (e.g., Ang II-infused mice, DOCA-salt rats, SHR) provides the most relevant support, demonstrating upregulation of Piezo1/TRPV4 and immune cell infiltration in the context of elevated systemic BP [12,15,16,26,45,89]. Second, studies in pulmonary hypertension offer valuable mechanistic insights into mechano-immune interactions in a high-pressure vascular bed, but translation to the systemic circulation requires caution due to differences in hemodynamics and vascular biology [50,51,90]. Third, observations from non-hypertensive mechanical overload models (e.g., transverse aortic constriction, podocyte stretch in vitro) establish biological plausibility by showing that isolated mechanical stress can drive inflammation, yet these models lack the systemic neurohumoral milieu of hypertension [46,52,91]. Fourth, indirect evidence extrapolated from other diseases (cancer, developmental biology, fibrosis) provides important mechanistic hypotheses (e.g., YAP/TAZ signaling) but remains several steps removed from the hypertensive context [21,39,55,78,79]. Throughout this review, we explicitly indicate which category of evidence underpins each statement, and we acknowledge that the integrative model, while mechanistically coherent, currently rests on a foundation of convergent preclinical data rather than definitive human causal proof.

### 6.1. The Loop Model

At its core, the mechano-immune activation loop can be conceptualized as a cascade of events that spirals from hemodynamic derangement to immune effector function to structural vascular change and back to hemodynamic derangement. The following scheme illustrates this proposed progression:

Step 1: Blood pressure elevation. Hypertension, whether driven by genetic predisposition, dietary factors, neurohumoral activation, or renal dysfunction, initially elevates luminal pressure. This primary hemodynamic change is the trigger that sets the entire loop in motion [7]. Importantly, once the loop is established, blood pressure elevation becomes both the input that drives mechanical stress and the output of the vascular remodeling it induces.

Step 2: Aberrant mechanical forces. Elevated blood pressure translates directly into abnormal mechanical forces across the vascular wall: shear stress on the endothelium increases in magnitude and becomes disturbed in direction; cyclic stretch of VSMCs and adventitial fibroblasts increases in amplitude and frequency; and hydrostatic pressure rises throughout the wall, particularly affecting high-pressure beds such as the renal microcirculation [41,45,46]. These forces are not uniform—they vary by vessel segment, creating regional hotspots of mechano-immune activation.

Step 3: Mechanosensitive molecule activation. These aberrant forces engage the mechanosensory apparatus described in Section 3: Piezo1 and TRPV4 are opened by membrane tension and stretch, while integrins are activated by increased ECM stiffness and force transmission through focal adhesions [12,14,19,22]. However, it is now clear that these systems are not independent. Emerging evidence reveals that Piezo1 itself can be recruited to focal adhesions in a force-dependent manner, where it directly drives adhesion dynamics and calcium signaling to promote cell spreading and adhesion maturation [68]. This Piezo1-integrin crosstalk is bidirectional: integrin activation can modulate Piezo1-dependent inflammatory signaling, and Piezo1-mediated signals can in turn regulate cytoskeletal tension and ECM remodeling, creating an integrated mechanosensing hub at the cell–matrix interface [92]. The downstream signals branch into Ca^2+^-dependent (calcineurin-NFAT, NLRP3), FAK-dependent (MAPK, NF-κB), and Hippo-dependent (YAP/TAZ) pathways [23,30].

Step 4: Immune cell activation. These signals do not remain confined to vascular wall cells; they directly activate infiltrating and tissue-resident immune cells (Section 5) [91]. T cells polarize toward the Th17 lineage with increased IFN-γ production; macrophages shift toward the M1 pro-inflammatory phenotype with elevated IL-1β secretion; and neutrophils undergo enhanced NETosis [26]. This immune activation is not a passive consequence of hypertension but an active, mechanically driven process.

Step 5: Inflammatory effector outputs. The activated immune cells secrete a cocktail of inflammatory mediators: IL-17A impairs endothelial function and promotes neutrophil recruitment; IL-1β stimulates VSMC proliferation and collagen synthesis; IFN-γ activates macrophages and further upregulates adhesion molecules; and ROS and matrix metalloproteinases (MMPs) degrade elastin, compromising the structural integrity of the vessel wall [22,85,87]. These effectors collectively shift the vascular wall from a homeostatic to a pro-remodeling state [93,94,95].

Step 6: Vascular remodeling and stiffening. The cumulative effect of these inflammatory outputs is vascular remodeling: VSMC phenotypic switching from contractile to synthetic or osteogenic phenotypes, excessive ECM deposition (collagen I/III), fragmentation of elastin fibers, and increased cross-linking of matrix proteins [22,23]. The vessel wall becomes thicker, stiffer, and less compliant. Arterial stiffness is both a structural marker of hypertensive damage and a powerful amplifier of blood pressure.

Step 7: Loop closure. Stiffened arteries increase pulse pressure and pulse wave velocity, which in turn elevate systolic blood pressure and further augment mechanical stress on the endothelium and VSMCs [49]. This closes the loop: the output of vascular remodeling feeds back to the input of mechanical force, creating a self-sustaining cycle that progressively worsens hypertension and its associated end-organ damage. Critically, this closed loop is not merely a unidirectional cascade but a truly bidirectional circuit: the output of vascular remodeling (Step 6) directly feeds back to augment the input of mechanical force (Step 2), while the inflammatory mediators (Step 5) simultaneously sensitize the mechanosensory machinery (Step 3), lowering the threshold for future activation. This bidirectional nature distinguishes the loop from a simple linear pathway and underpins its self-perpetuating character.

In vivo support for this step comes from hypertensive models: uninephrectomised aldosterone-infused mice show Piezo1 upregulation in podocytes correlating with injury markers [45]; spontaneously hypertensive rat and Ang II-infused rats exhibit Piezo2 downregulation in nodose ganglia with impaired baroreflex [15]; Deoxycorticosterone acetate (DOCA)-salt hypertensive rats show left ventricular Piezo1/2 upregulation [89]. TRPV4 expression and activity are elevated in hypertensive vasculature, contributing to endothelial dysfunction [16].

### 6.2. Molecular Nodes of the Loop

The mechano-immune loop is not a diffuse, non-specific process; it depends on a series of discrete molecular nodes that perform defined functions at specific positions within the circuit. Targeting these nodes offers the potential to interrupt the loop therapeutically (Table 3).

Mechanosensing node (Piezo1, TRPV4). These channels serve as the loop’s entry points. They are directly gated by physical forces—shear, stretch, or pressure—and are expressed both on vascular cells and on the immune cells that interact with the vasculature. Their activation does not require intermediary biochemical signals, making them the most proximal and rapid responders to hypertensive mechanical stress [12,14]. Variation in their expression levels (e.g., Piezo1 upregulation in hypertensive podocytes [45]) directly modulates the gain of the loop.

Ca^2+^ node (Calcineurin, CaMK). Ca^2+^ influx through Piezo1 and TRPV4 is the first amplification step [96]. Through calcineurin, it drives NFAT-dependent cytokine transcription; through CaMK, it activates CREB for cell survival and metabolic adaptation. This node is a branch point, distributing a single mechanical input into multiple downstream transcriptional programs.

Transcriptional node (NFAT, NF-κB, YAP). These three transcription factors/co-activators integrate mechanical signals (Ca^2+^, integrin-FAK, Hippo) and coordinate the expression of a broad inflammatome. NFAT drives IL-2, IFN-γ, and IL-17A in T cells; NF-κB drives IL-6, TNF-α, and adhesion molecules in macrophages and endothelial cells; YAP/TAZ amplifies NF-κB activity and promotes ECM-remodeling genes [23,26]. Their simultaneous engagement ensures that mechanical forces evoke a full-spectrum inflammatory response, not merely a single cytokine.

Inflammatory effector node (IL-17A, IL-1β, IFN-γ). These cytokines and mediators are the functional outputs of immune activation. IL-17A recruits neutrophils and impairs endothelial NO production [76]; IL-1β drives VSMC proliferation and induces further cytokine release [97]; IFN-γ activates macrophages and upregulates MHC expression [98]. Their presence in hypertensive tissues has been consistently documented, and their neutralization attenuates hypertensive vascular injury in animal models [26,85,87].

Vascular remodeling node (MMPs, transforming growth factor-beta (TGF-β), Collagen). This node is the structural consequence of the loop. MMPs degrade elastin and ECM components; TGF-β stimulates fibroblast-to-myofibroblast differentiation and collagen synthesis; and excess collagen cross-linking increases wall stiffness. The mechanical changes produced at this node—increased stiffness and pulse pressure—directly feed back to augment the input signal (mechanical forces) that drives the entire loop [22,47,49].

### 6.3. Positive Feedback Characteristics of the Loop

The mechano-immune activation loop is defined by its positive feedback architecture: the output of the loop (vascular stiffening and elevated blood pressure) enhances its own input (mechanical stress). This section analyses the quantitative and qualitative properties that arise from this architecture.

Mechanical force ↑ → Immune activation → Vascular stiffening → Mechanical force further ↑. This is the fundamental positive feedback cycle. Each complete circuit amplifies the initial signal, converting a modest blood pressure elevation into a progressive, self-accelerating hypertensive state. The loop does not simply maintain hypertension; it escalates it.

Loop gain: the amplification factor per cycle. The gain of the loop refers to the degree to which one pass through the circuit amplifies the original signal. If a 10 mmHg blood pressure elevation produces, through mechanical stress and immune activation, a vascular stiffening that raises blood pressure by an additional 5 mmHg, the gain is 0.5. If gain exceeds 1, the loop is explosive—each cycle produces a larger increment than the previous one. In clinical hypertension, gain is likely >1 in the early phases of progression, driving the rapid escalation of blood pressure and vascular damage, but may approach 1 in the established phase as compensatory mechanisms (e.g., baroreflex, renal pressure-natriuresis) exert counter-regulation. The gain is modulated by factors such as Piezo1 expression level, immune cell infiltrate density, and ECM cross-linking rates [12,29,99].

Loop threshold: when does the loop transition from adaptive to pathological? Under physiological conditions, low-grade mechanical forces and transient immune responses contribute to vascular homeostasis—endothelial NO production, VSMC tone regulation, and tissue repair. However, when blood pressure exceeds a certain threshold (which may vary individually depending on genetic and environmental factors), the scale of mechanical stress overwhelms homeostatic protective mechanisms. At this threshold, the positive feedback gain becomes >1, and the system transitions from adaptive to pathological. Factors that lower this threshold include aging (which increases baseline arterial stiffness [49]), salt sensitivity (which increases pressure-natriuresis gain), and genetic predispositions affecting mechanosensor expression.

Self-sustaining nature and irreversibility. Once the loop gain exceeds 1 and the threshold is crossed, the loop can become self-sustaining. Even if the original trigger (e.g., a transient volume overload) resolves, the vascular remodeling and immune activation persist through the loop’s own internal dynamics. This self-sustaining property is a key reason why established hypertension often becomes refractory to single-agent therapy and requires combination approaches [100]. The structural changes—collagen deposition, elastin fragmentation, arterial stiffening—are not easily reversible, and the immune infiltrate can persist as tissue-resident memory cells that re-activate upon subsequent mechanical stimuli [101,102]. This irreversibility underscores the importance of early intervention to prevent loop establishment.

Definitive genetic proof comes from conditional knockout approaches: endothelial-specific Piezo1 deletion impairs shear-induced vasodilation and elevates blood pressure, as Piezo1 is required for flow-dependent NO production [14]. Transverse aortic constriction (TAC) models demonstrate that local mechanical overload alone—isolated from systemic neurohumoral activation—is sufficient to drive macrophage/T cell infiltration and pro-inflammatory cytokine expression, and this infiltration is attenuated by mechanosensitive channel inhibitors. Pharmacological inhibition with GsMTx4 reduces proteinuria in hypertensive nephropathy [45]; TRPV4 antagonists (GSK2193874, HC-067047) attenuate vascular inflammation and fibrosis [16]; P2X7 receptor knockout reduces Ang II-induced hypertension and immune activation [103]; Axl inhibition reduces renal and vascular inflammation [104]. Collectively, these in vivo interventions demonstrate that blocking mechanosensitive channels can interrupt the loop and mitigate hypertensive damage.

### 6.4. Crosstalk of the Mechano-Immune Loop with Other Hypertensive Pathways

The mechano-immune loop does not operate in isolation. It intersects with, amplifies, and is amplified by other well-known hypertensive mechanisms, forming a broader network of interacting positive feedback circuits.This intricate crosstalk reinforces the view that the loop is not a one-way street but a dynamic, bidirectional interface where mechanical and biochemical signals constantly modulate each other.

Intersection with the Ang II–NF-κB–IL-6–Th17 axis. The renin–angiotensin–aldosterone system (renin–angiotensin–aldosterone system (RAAS)) is a central driver of hypertension [105]. Angiotensin II (Ang II) promotes NF-κB-dependent IL-6 production from macrophages, which in turn stimulates Th17 differentiation and angiotensinogen expression in renal proximal tubular cells via STAT3 [87]. The mechano-immune loop intersects with this pathway at multiple levels: (a) mechanical stress upregulates angiotensinogen expression and AT1 receptor density, enhancing Ang II signaling; (b) Ang II itself can sensitize Piezo1 channels, lowering their activation threshold—a possibility supported by the observation that Ang II and mechanical stretch synergistically activate VSMC inflammatory responses [22]; and (c) IL-17A from mechanically activated T cells promotes renal Ang II production, further driving RAAS activation. This creates a double positive feedback: mechanical forces drive immune cells that produce IL-17, which enhances Ang II, which further potentiates mechanical sensitivity.

Synergy with the high salt–NLRP3–IL-1β axis. High dietary salt is a well-established risk factor for hypertension [106,107]. Salt activates the NLRP3 inflammasome in macrophages and renal cells, leading to IL-1β production and sodium retention. The mechano-immune loop synergizes with this axis: (a) mechanical stretch itself is a potent NLRP3 activator through TRPV4-mediated Ca^2+^ influx, providing a second, parallel input to IL-1β release [16]; (b) salt-induced water retention elevates blood pressure, thereby directly increasing mechanical stress—the loop’s input; and (c) IL-1β promotes ECM remodeling and vascular stiffness, feeding back to augment mechanical forces [46,85]. Thus, salt and mechanical stress are not independent risk factors but synergistic drivers of the same IL-1β-dependent inflammatory cascade.

Connection with the sympathetic–immune loop. The sympathetic nervous system (sympathetic nervous system (SNS)) is hyperactive in hypertension and directly modulates immune cell function through β-adrenergic receptors [108]. Sympathetic nerve terminals in the kidney and spleen release noradrenaline, which activates immune cells, promotes cytokine production, and mobilizes leukocytes. The mechano-immune loop connects to the SNS via: (a) mechanical stress activates renal afferent nerves, which increase central sympathetic outflow—a reflex that amplifies blood pressure; (b) sympathetic outflow itself elevates blood pressure, adding to mechanical stress; and (c) catecholamines enhance Piezo1 expression and sensitivity on immune cells, lowering the mechanical threshold for their activation [108,109]. This creates a tripartite loop: mechanical stress → SNS activation → blood pressure elevation + immune sensitization → further mechanical stress.

**Figure 3:**
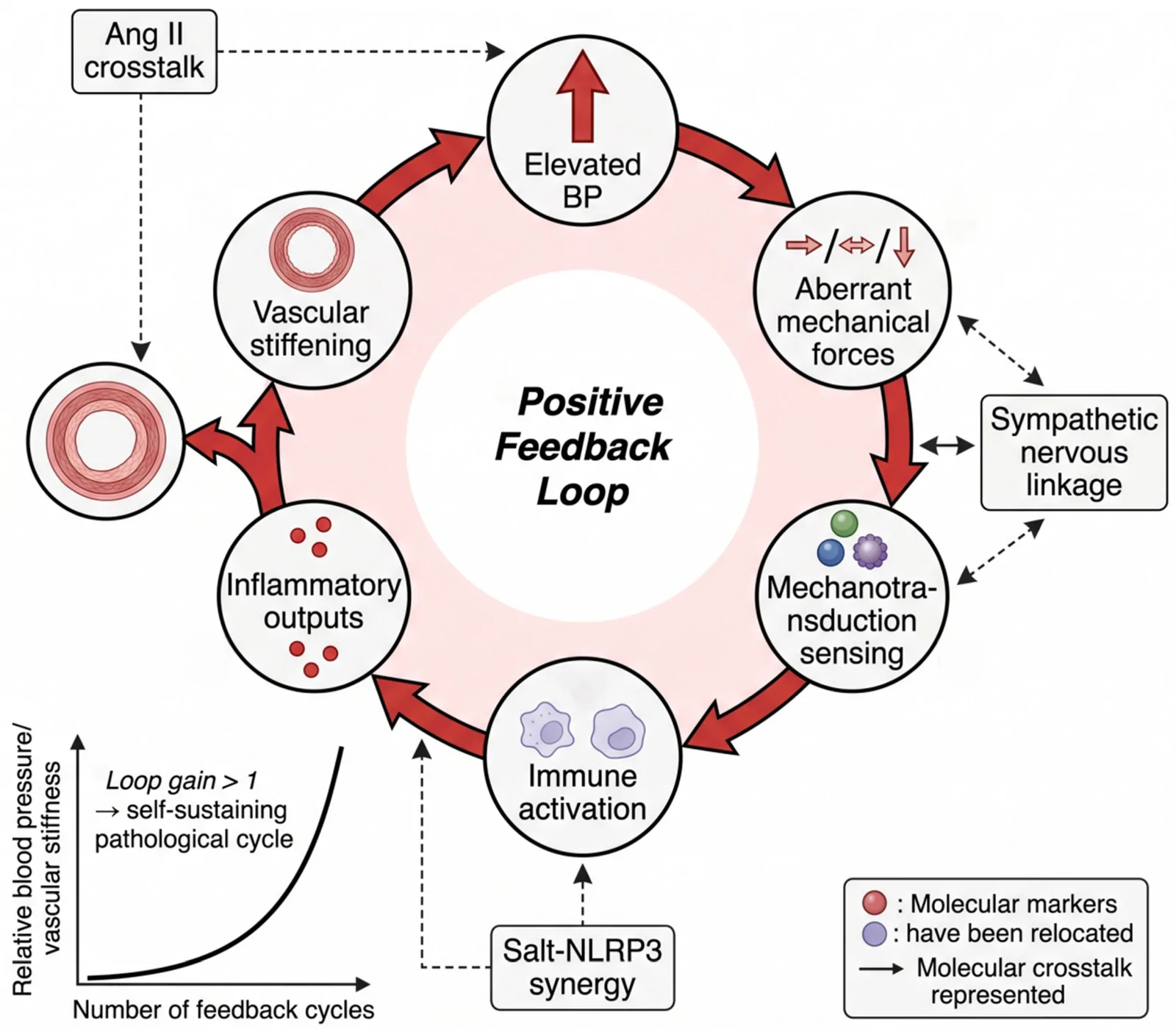
The mechano-immune-vascular activation loop: a bidirectional, self-sustaining positive feedback circuit in hypertension. This schematic integrates the molecular, cellular, and organ-level components of the proposed mechano-immune-vascular loop into a unified model. The loop operates in distinct mechano-immune microenvironments, contributing to glomerulosclerosis and albuminuria (kidney), endothelial dysfunction and stiffening (large arteries), hypertrophy and fibrosis (heart), and blood–brain barrier disruption and neuroinflammation (brain). Figure 3 was created with Figdraw (www.figdraw.com). Abbreviations: NLRP3, NLR family pyrin domain containing 3; Ang II, angiotensin II; BP, blood pressure.

Convergence on shared effectors. Ultimately, all these pathways converge on the same downstream effectors—NF-κB, IL-17A, IL-1β, ROS, and TGF-β—that drive vascular remodeling [110]. The mechano-immune loop thus acts not as a standalone pathway but as an integrated hub that amplifies and is amplified by RAAS, salt, and SNS pathways, creating a robust, multi-input positive feedback network that sustains hypertension and its complications (Figure 3) [90,111].

## 7. Organ-Specific Mechano-Immune Microenvironments

### 7.1. Kidney

The kidney is uniquely vulnerable to hypertension, as its microcirculation operates under high hydrostatic pressure and experiences dynamic changes in flow and shear. These mechanical may stresses engage mechanosensors on resident and infiltrating cells, driving local inflammation, glomerular injury, and fibrosis [112].

Glomerular high hydrostatic pressure → podocytes/mesangial cells → Piezo1 → inflammation. Podocytes are terminally differentiated cells that form the outer layer of the glomerular filtration barrier. They are subjected to hydrostatic pressure and cyclic stretch transmitted from the capillary loop. In hypertensive nephropathy, Piezo1 expression is upregulated in podocytes, mesangial cells, and distal tubular cells, correlating with the injury markers plasminogen activator inhibitor-1 (PAI-1) and SGK1. Mechanical stretch of Piezo1-expressing podocytes activates Rac1, upregulates these markers, and promotes cytoskeletal remodeling and podocyte foot process effacement. These effects are reversed by the Piezo1 blocker GsMTx4 [45]. Mesangial cells, which support the glomerular capillary tuft, also respond to mechanical stretch through Piezo1 and integrin-mediated FAK activation, contributing to mesangial matrix expansion and glomerulosclerosis [46].

Tubular high flow → shear stress → tubular epithelium → immune signaling. The renal tubular epithelium is exposed to fluid shear stress from the ultrafiltrate. In states of increased glomerular filtration or compensatory hyperfiltration, elevated tubular flow enhances shear stress on the luminal membrane, activating mechanosensors such as primary cilia and integrins. This induces tubular epithelial cells to produce chemokines (MCP-1, regulated on activation, normal T cell expressed and secreted) and adhesion molecules that recruit immune cells into the interstitium. In co-culture experiments, angiotensin II-treated macrophages secrete IL-6, which stimulates renal proximal tubular cells to produce angiotensinogen via the Janus kinase (JAK)-STAT pathway [87]. This bidirectional crosstalk between tubular epithelium and macrophages, likely enhanced by mechanical stress, contributes to the tubulointerstitial inflammation characteristic of hypertensive nephropathy.

Renal interstitial high pressure → resident macrophages → NLRP3. The renal interstitium experiences elevated hydrostatic pressure in hypertension, which directly compresses interstitial macrophages and DCs. These cells, which are highly mechanosensitive, respond by upregulating NLRP3 inflammasome activity, leading to caspase-1-dependent IL-1β and IL-18 secretion. IL-1β promotes fibroblast activation, collagen deposition, and further macrophage recruitment, creating a self-sustaining inflammatory niche. The renal pelvis and papilla, where interstitial pressures are highest, are particularly susceptible to this mechano-inflammatory response [85].

### 7.2. Large Arteries

Large elastic arteries, such as the aorta and carotid arteries, are the primary conduits for blood from the heart and are exposed to the highest pulsatile forces. Their remodeling in hypertension not only raises pulse pressure but also sets the stage for accelerated atherosclerosis and aneurysm formation.

Endothelial high shear stress → Piezo1 → adhesion molecules → immune cell recruitment. The endothelium lining the aorta is constantly exposed to shear stress. In hypertension, shear stress increases in magnitude and becomes disturbed at bifurcations and curvatures. Piezo1 on endothelial cells senses these changes, and disturbed shear upregulates adhesion molecules (ICAM-1, VCAM-1, endothelial selection), promoting the capture and transmigration of monocytes and T lymphocytes [41]. Computational fluid dynamics simulations of pulmonary arterial trees with ventricular septal defects show that large defects induce wall shear stress >100 dyn/cm^2^ in small-diameter vessels, a threshold linked to dysfunctional mechanotransduction [51]. In hypertensive large arteries, similar high-shear hotspots trigger local endothelial activation and immune cell homing, initiating vascular inflammation.

Smooth muscle high cyclic stretch → TRPV4 → M1 polarization. VSMCs in the medial layer experience cyclic stretch with each cardiac cycle. In hypertension, the amplitude and frequency of this stretch are augmented, activating TRPV4 and other mechanosensors. TRPV4-mediated Ca^2+^ influx promotes VSMC proliferation and also drives the polarization of infiltrating and tissue-resident macrophages toward the pro-inflammatory M1 phenotype. This is further amplified by the release of chemokines from stretched VSMCs, which recruit additional monocytes and perpetuate M1 dominance [22].

Arterial stiffening → pulse wave velocity ↑ → mechanical forces further ↑. The structural consequences of vascular inflammation—elastin fragmentation, collagen deposition, and VSMC hypertrophy—increase arterial stiffness, which increases pulse wave velocity (pulse wave velocity (PWV)). Elevated PWV means that the pressure wave returns to the aorta earlier, augmenting systolic pressure and further increasing the mechanical stress on the vessel wall. This is the classic positive feedback loop at the organ level: inflammation stiffens the artery, and stiffness increases the mechanical load, which promotes more inflammation [49]. This vicious cycle is central to the progression of isolated systolic hypertension in the elderly and is a major driver of cardiovascular events [110].

### 7.3. Heart

The heart experiences pressure overload in hypertension, leading to concentric hypertrophy, fibrosis, and eventually heart failure. Mechano-immune interactions in the myocardium play a crucial role in this transition.

Pressure overload → cardiomyocyte Piezo1 → hypertrophy + inflammation. Left ventricular pressure overload, resulting from elevated aortic pressure, increases wall stress and triggers hypertrophic responses in cardiomyocytes. Piezo1 is expressed on cardiomyocytes and is activated by increased membrane tension. Piezo1-mediated Ca^2+^ influx activates calcineurin-NFAT, which drives the transcription of hypertrophic genes (e.g., atrial natriuretic peptide (ANP), B-type natriuretic peptide (BNP)) and also upregulates inflammatory cytokines such as TNF-α and IL-6. The in vitro platforms for studying cardiomyocyte mechanobiology have shown that ECM stiffness and mechanical stretch regulate cardiac cell function, and aberrant mechanotransduction contributes to hypertensive heart disease [113]. In rats with high-fat diet and DOCA-salt hypertension, Piezo1 and Piezo2 expression in the left ventricle is significantly upregulated, reflecting the adaptive mechanosensory response to pressure overload.

High filling pressure → atrial stretch → immune cell infiltration. As left ventricular compliance decreases with hypertrophy and fibrosis, left atrial pressure rises, stretching the atrial wall. This mechanical stretch activates resident fibroblasts and endothelial cells to secrete chemokines (e.g., MCP-1, IL-8), recruiting macrophages and T cells into the atrial interstitium [114]. The resulting atrial inflammation, along with the mechanical stress itself, promotes atrial fibrosis and remodeling, predisposing to atrial fibrillation—a common comorbidity of hypertensive heart disease. Cardiac microvascular endothelial activation and oxidative stress, driven by mechanical forces and inflammation, contribute to microvascular rarefaction, ischemia, and fibrosis in heart failure with preserved ejection fraction (HFpEF) [115].

Fibrosis and diastolic dysfunction. The inflammatory cells recruited to the heart produce TGF-β, which stimulates fibroblast-to-myofibroblast differentiation and excessive collagen deposition. This interstitial fibrosis impairs diastolic relaxation, increases ventricular stiffness, and further elevates filling pressures, closing the mechano-immune loop at the cardiac level [55].

### 7.4. Brain

The cerebral circulation is uniquely susceptible to the adverse effects of hypertension, because the brain has limited autoregulatory capacity and is highly dependent on constant perfusion [116].

Hypertension → cerebral microvascular shear stress → blood–brain barrier. The cerebral microvessels maintain the blood–brain barrier (blood–brain barrier (BBB)), which is essential for neural homeostasis. In hypertension, elevated shear stress and cyclic stretch damage the endothelium of penetrating arterioles and capillaries, leading to BBB disruption. This allows plasma proteins (e.g., fibrinogen, albumin) and immune cells to enter the brain parenchyma, initiating perivascular inflammation. The BBB breakdown is an early event in hypertensive cerebral small vessel disease and correlates with cognitive decline [117,118].

Cerebral mechanical forces and neuroinflammation. In addition to the direct effects on vessels, mechanical forces within the brain parenchyma—generated by pulsatile pressure transmission through the arterial tree—activate microglia, the brain’s resident immune cells. Microglia express Piezo1 and TRPV4, and their activation by mechanical stretch induces the production of pro-inflammatory cytokines (TNF-α, IL-1β) and reactive oxygen species [119,120]. This microglial activation contributes to neuronal injury, synaptic dysfunction, and neurovascular uncoupling, further impairing cerebral blood flow regulation.

Implications for cognitive impairment. Chronic neuroinflammation, driven by the mechano-immune loop, is increasingly recognized as a contributor to vascular cognitive impairment and dementia. Hypertension accelerates white matter lesions, silent infarcts, and brain atrophy, all linked to inflammation. Immune system activation and cognitive impairment are closely intertwined in arterial hypertension, with chronic inflammation disrupting the blood–brain barrier, leading to neurovascular dysfunction [117]. Understanding the role of mechanical forces in this process may open new avenues for preventing hypertensive encephalopathy (Table 4).

### 7.5. Systemic Integration: Organ Crosstalk in the Mechano-Immune-Vascular Loop

While the preceding sections have detailed organ-specific manifestations of the mechano-immune-vascular loop, hypertension is fundamentally a systemic disease in which these local processes are not isolated but intimately interconnected through hemodynamic, neurohumoral, and inflammatory pathways. Understanding these inter-organ crosstalks is essential for appreciating how the loop operates as a distributed network rather than a collection of independent, tissue-autonomous circuits.

Kidney–Vascular Axis. The kidney plays a dual role as both a source and a target of mechano-immune activation. Renal injury driven by high hydrostatic pressure and Piezo1-mediated podocyte damage promotes sodium retention and volume expansion, which directly elevates systemic blood pressure and increases mechanical stress on large arteries [112]. Conversely, arterial stiffening—driven by the vascular loop in the aorta and carotids—increases pulse pressure, which is transmitted into the renal microcirculation, exacerbating glomerular hypertension and perpetuating kidney injury [49]. This bidirectional kidney–vascular interaction creates a vicious cycle: stiff arteries damage the kidney, and the damaged kidney raises blood pressure, which stiffens arteries further.

Heart–Brain Axis. Cardiac dysfunction and cerebral pathology are similarly intertwined. Pressure overload-induced left ventricular hypertrophy and diastolic dysfunction elevate left atrial and pulmonary venous pressures, which are transmitted to the cerebral venous system, impairing cerebral venous drainage and potentially exacerbating blood–brain barrier dysfunction [117,118]. Concurrently, the neuroinflammation driven by cerebral microglial activation (Section 7.4) promotes sympathetic outflow from the brainstem, which increases cardiac afterload and worsens cardiac remodeling [119].

Renal–Cardiac Axis (Cardiorenal Syndrome Type 2). In the hypertensive context, chronic kidney disease and heart failure frequently coexist and reinforce each other. The systemic inflammation and oxidative stress originating from the hypertensive kidney—driven by the renal mechano-immune loop—directly promote cardiac fibrosis and diastolic dysfunction through circulating cytokines (IL-1β, IL-6, TNF-α) and activated immune cells that home to the myocardium [55,115]. This “inflammatory crosstalk” is superimposed on the classical hemodynamic effects (volume overload, uremic toxins), making the renal–cardiac axis a critical amplifier of the overall loop.

Common Immune Cell Traffic as a System-Wide Connector. Crucially, the immune cells activated within each organ are not confined to their tissue of origin. T cells and macrophages primed by mechanical stress in the kidney or aorta can recirculate and infiltrate other vascular beds, carrying their mechano-imprinted inflammatory phenotype to distant sites [101,102]. This systemic dissemination of activated immune cells provides a mechanistic link that synchronizes the loop across multiple organs, transforming local injury into a systemic inflammatory state.

Implications for Disease Progression and Therapy. The existence of these inter-organ crosstalks has three important clinical implications. First, it explains why end-organ damage in hypertension is often multiorgan and coexisting—e.g., the frequent combination of hypertensive nephropathy, heart failure, and cognitive decline in the same patient. Second, it suggests that interventions targeting a single organ may be insufficient; effective therapy may need to break the loop at multiple sites simultaneously or at a systemic level (e.g., by targeting common immune mediators or reducing global arterial stiffness). Third, it highlights that organ-specific biomarkers (e.g., urine albumin for the kidney, pulse wave velocity for arteries, N-terminal pro-B-type natriuretic peptide (NT-proBNP) for the heart) should be interpreted collectively, as they reflect different facets of a unified systemic process. Future studies should prioritize the characterization of this systemic mechano-immune network, using multi-organ imaging, immune cell tracking, and computational modeling to map the spatiotemporal dynamics of loop activation across the whole organism.

## 8. Therapeutic Implications: Targeting the Mechano-Immune Loop

If the proposed mechano-immune activation loop is validated in future studies, its delineation could identify multiple nodes amenable to therapeutic intervention. Strategies may target the mechanosensitive ion channels themselves, exploit the mechano-immunomodulatory effects of existing antihypertensive drugs, modify the mechanical environment, or combine these approaches. However, each strategy faces significant challenges, and it is important to recognize that all therapeutic implications discussed here are speculative, based on the conceptual model rather than on proven clinical efficacy.

### 8.1. Mechanosensitive Channels as Drug Targets

Pharmacological modulation of mechanosensitive ion channels holds promise for interrupting the loop at its entry point. Several tool compounds exist, though clinical development remains in early stages (Table 5).

Piezo1 inhibitors GsMTx4. GsMTx4 is a spider venom-derived peptide that inhibits Piezo1 and other mechanosensitive channels with moderate selectivity. In hypertensive nephropathy models, GsMTx4 reversed mechanical stretch-induced podocyte injury and reduced proteinuria [45]. It has also been shown to reduce vascular inflammation in some models. However, GsMTx4 is a peptide with limited oral bioavailability and potential off-target effects, making it unsuitable for chronic systemic therapy. Efforts are underway to develop small-molecule Piezo1 inhibitors with improved pharmacokinetics.

Piezo1 agonists (Yoda1). Yoda1 is primarily a research tool to study Piezo1 function. It has vasodilatory effects (through endothelial NO release) and may have therapeutic potential in some vascular disorders, but its pro-inflammatory effect on immune cells could exacerbate hypertension [121]. Therefore, agonists are unlikely to be useful in treating hypertension unless highly cell-type-specific targeting is achieved.

TRPV4 antagonists. GSK2193874 and HC-067047 are potent TRPV4 antagonists that have shown efficacy in reducing pulmonary hypertension and cardiac fibrosis in preclinical models [13]. They block Ca^2+^-dependent NLRP3 inflammasome activation in macrophages, attenuating IL-1β production. TRPV4 antagonists are being investigated in clinical trials for other indications (e.g., heart failure, chronic cough), and repurposing for hypertension could be feasible if selectivity is maintained.

TRPC6 inhibitors. BI-749327 is a selective TRPC6 inhibitor that reduced hypertension and renal injury in models of salt-sensitive hypertension. By blocking TRPC6 in VSMCs, it limits myogenic tone and prevents pressure-induced vasoconstriction, while also dampening the mechanosensitive Ca^2+^ signals that promote vascular remodeling [18].

Integrin inhibitors. Integrin antagonists, such as cilengitide (αvβ3/αvβ5 inhibitor), have been explored in oncology but not yet in hypertension. Given the central role of integrins in mechanotransduction and immune adhesion, targeted integrin inhibition could reduce VSMC phenotypic switching and leukocyte infiltration [19]. However, the broad expression of integrins raises concerns about bleeding and wound-healing complications.

Human evidence for the loop remains largely correlational. Plasma cyclophilin-A (CyP-A), a marker of vascular inflammation, is elevated in hypertensive patients and correlates with systolic/diastolic BP [122]; UK Biobank data show P2RX7 polymorphisms associated with diastolic BP elevation [103]; anti-Hsp60 antibodies correlate with hypertension severity and target organ damage [123]. In atherosclerotic plaques from hypertensive patients, endothelial erosion localizes to low-shear regions and is characterized by TLR signaling [42]. Multi-biomarker panels (NT-proBNP, TGF-β, CT-1, cystatin C (CysC)) improve heart failure prediction beyond NT-proBNP alone [124], suggesting mechanical and immune markers jointly reflect disease severity. However, no prospective interventional study has tested whether pharmacological modulation of Piezo1 or TRPV4 affects BP or inflammatory markers in patients; no study has directly measured immune cell mechanosensitivity from hypertensive patients. Thus, while correlational human data are suggestive, causal evidence is absent.

### 8.2. Mechano-Immunomodulatory Effects of Existing Antihypertensive Drugs

Many standard antihypertensive agents, while primarily designed to lower blood pressure, also exert direct or indirect effects on the mechano-immune loop.

Angiotensin-converting enzyme inhibitors (ACEIs) and angiotensin II receptor blockers (ARBs). Beyond lowering blood pressure—and thus reducing mechanical stress—ACEIs and ARBs possess anti-inflammatory properties. They reduce NF-κB activation, decrease cytokine production, and attenuate immune cell infiltration [125]. The reduction in angiotensin II levels also lowers the sensitization of Piezo1 to mechanical stretch, potentially raising the threshold for mechano-immune activation. In hypertensive patients, treatment with ACEIs/ARBs reduces circulating IL-6 and C-reactive protein (CRP) levels.

Calcium channel blockers (CCBs). CCBs lower blood pressure by blocking L-type Ca^2+^ channels in VSMCs. They also inhibit Ca^2+^ influx in immune cells, including T cells and macrophages, although this is not their primary mechanism [125]. By reducing intracellular Ca^2+^, CCBs may blunt calcineurin-NFAT signaling downstream of Piezo1/TRPV4, thus dampening immune activation. However, this effect is non-selective and may also impair beneficial immune functions.

Mineralocorticoid receptor (MR) antagonists. MR antagonists (e.g., spironolactone, eplerenone) not only lower blood pressure through natriuretic effects but also have robust anti-fibrotic and anti-inflammatory actions [126]. They reduce ECM deposition, improve vascular compliance, and decrease macrophage infiltration. By improving the mechanical properties of the vessel wall, they indirectly reduce mechanical stress and break the loop at the feedback level.

Statins. Although not primarily antihypertensive, statins are widely used in hypertensive patients with dyslipidemia. They reduce vascular inflammation, lower MMP activity, and improve endothelial function, thereby attenuating both the inflammatory effector node and the vascular remodeling node of the loop [127,128].

### 8.3. Interventions Targeting the Mechanical Environment

Modifying the mechanical environment itself—rather than the molecular sensors—offers an alternative approach to disrupting the loop.

Strict blood pressure control. The most direct way to reduce mechanical stress is to lower blood pressure effectively. Targeting systolic BP to <130 mmHg (as recommended by recent guidelines [129]) reduces the magnitude and duration of shear stress and cyclic stretch, thereby decreasing the input to the mechano-immune loop. Clinical trials have shown that intensive BP lowering reduces inflammatory biomarkers and slows vascular remodeling [130]. However, the mechano-immune loop may become self-sustaining even after BP normalization, especially if significant arterial stiffening and immune memory have already developed.

Strategies to improve vascular compliance. Beyond lowering BP, therapies that directly improve arterial compliance—such as inhibitors of advanced glycation end-products (AGEs), agents that promote elastin regeneration, and drugs that reduce collagen cross-linking (e.g., relaxin, serine protease inhibitors)—could reduce pulse pressure and pulse wave velocity, thereby breaking the feedback from stiffness to mechanical stress [131].

Adaptive effects of exercise training. Regular aerobic exercise increases laminar shear stress and improves endothelial function through mechano-adaptive responses. Exercise upregulates endothelial NO synthase, reduces oxidative stress, and promotes anti-inflammatory immune profiles, potentially lowering the gain of the mechano-immune loop. Exercise training also reduces arterial stiffness over time. While not a direct pharmacological intervention, lifestyle modification that alters the mechanical environment is a cornerstone of hypertension management.

### 8.4. Challenges in Targeting the Mechano-Immune Loop

Despite the theoretical promise of targeting the mechano-immune loop, several obstacles hinder clinical translation.

Broad expression of mechanosensitive molecules → non-specific effects. Piezo1, TRPV4, and integrins are expressed in many cell types throughout the body, including essential organs (heart, kidney, brain) and normal immune cells [4]. Systemic inhibition could impair physiological mechanosensing, such as baroreflex function, renal autoregulation, and immune surveillance [75,132]. For example, Piezo1 is required for shear-induced vasodilation; inhibiting it could paradoxically worsen endothelial function. Achieving cell-type-specific targeting—e.g., using antibody-drug conjugates or cell-targeted nanoparticles—is a major challenge that remains unresolved.

Redundancy in the loop nodes. The loop has multiple molecular entry points (Piezo1, TRPV4, integrins) and multiple downstream pathways (Ca^2+^-NFAT, NF-κB, YAP/TAZ). Blocking a single node may be insufficient if other pathways compensate. For instance, inhibiting Piezo1 may not prevent TRPV4-mediated NLRP3 activation or integrin-dependent FAK signaling. Combination therapy targeting multiple nodes may be required, but this increases the risk of adverse effects.

Long-term immunosuppression risk. Chronic inhibition of the mechano-immune loop could impair host defense against infections and possibly increase cancer risk [133,134,135]. The immune system relies on mechanosensing for proper trafficking, antigen presentation, and effector function. A careful risk-benefit assessment is essential, and strategies that transiently modulate the loop during high-risk periods (e.g., early hypertension) rather than lifelong suppression may be preferable.

Need for mechano-immune biomarkers to monitor efficacy. To individualize therapy, biomarkers that reflect the activity of the mechano-immune loop are needed [136]. Candidate markers include plasma cyclophilin-A (CyP-A) [122], NT-proBNP (reflecting cardiac mechanical stress) [124], and inflammatory cytokines such as IL-17A and IL-1β. However, these markers are not specific to the loop and may be influenced by many other factors. Multi-biomarker panels, combining a mechanical marker (e.g., CyP-A), an inflammatory marker (IL-6), and a remodeling marker (TGF-β), could provide a composite score for loop activity [137]. These panels require validation in prospective cohorts before they can guide therapy.

Reversibility. As noted earlier, the structural changes—arterial stiffening, fibrosis, and immune memory—may be only partially reversible, even if the loop is interrupted. This underscores the need for early intervention, before irreversible remodeling occurs [110]. Biomarkers that identify patients in the early, “reversible” phase of hypertension are urgently needed.

In summary, the mechano-immune loop presents a rich therapeutic landscape. While no loop-specific therapy is yet available for clinical use, existing antihypertensive drugs already exert beneficial effects that may in part reflect loop modulation. Future advances in targeted drug delivery, biomarker development, and combination regimens hold promise for translating mechano-immune insights into improved outcomes for hypertensive patients (Table 6).

## 9. Conclusions

In this review, we synthesize preclinical and correlational evidence to propose the Mechano-Immune-Vascular Activation Loop as an integrative framework for understanding mechanotransduction in hypertension. This model posits that elevated blood pressure generates aberrant mechanical forces—shear stress, cyclic stretch, and hydrostatic pressure—sensed by Piezo1, TRPV4, and integrins on vascular and immune cells [3,17]. This mechanotransduction may trigger inflammatory signaling through Ca^2+^-NFAT, NF-κB, YAP/TAZ, and MAPK pathways, promoting pro-inflammatory immune polarization (e.g., Th17 skewing, M1 macrophage activation) and cytokine secretion (IL-17A, IL-1β, IFN-γ) [20,37]. These mediators might drive VSMC phenotypic switching, extracellular matrix deposition, and arterial stiffening [21]. Such remodeling could elevate pulse pressure and pulse wave velocity, augmenting the mechanical forces that initiated the process, thus closing a positive feedback loop perpetuating hypertension and its complications [38,39].

This framework explains the role of low-grade inflammation in hypertension, the failure of blood pressure reduction alone to fully reverse vascular remodeling, and the link between hemodynamic derangement and organ damage. It suggests hypertension may be understood as a mechano-inflammatory disease.

However, current evidence remains largely associative and derived from reductionist in vitro systems and preclinical models. Definitive causal proof is lacking, as immune-cell-specific conditional knockouts of Piezo1 or TRPV4 have not been tested in hypertensive models, and human data are almost entirely correlational [41]. The field must move beyond phenomenological correlations and pharmacological tools with off-target effects to establish the hierarchical importance and redundancy of different mechanosensors. Furthermore, the interplay between mechanical signals and classical biochemical stimuli (Ang II, salt, cytokines) makes isolating a “pure” mechanical effect exceptionally difficult [42]. Future research should prioritize advanced in vivo models enabling cell-type-specific manipulation of mechanosensitive pathways to establish causality. Emerging technologies—organ-on-a-chip systems and high-resolution real-time imaging—will be crucial. Identifying mechano-immune biomarkers from hypertensive patients and testing whether pharmacological modulation of this loop affects clinical outcomes will be the ultimate test of translational relevance [14].

In conclusion, the Mechano-Immune-Vascular Activation Loop places bidirectional crosstalk between mechanotransduction and immunity at the heart of hypertension. While significant knowledge gaps remain, disrupting this potentially self-sustaining feedback cycle—rather than merely lowering blood pressure—opens new therapeutic avenues. Determining whether this self-amplifying cycle operates in human hypertension represents a critical frontier.

## Figures and Tables

**Table 1:** Distinct spatiotemporal profiles of mechanical stresses in hypertension: segment-specific force patterns, primary cellular targets, and key experimental models.

Force Type	Definition	Changes in Hypertension	Primary Site of Action	Key References	Study Sources
Shear stress	Frictional force exerted by blood flow on the vessel wall	Elevated magnitude + disturbed direction (turbulence)	Endothelial cells	[3,14,41,42,43]	Human (in vivo, in vitro); Mouse (in vivo, in vitro); Rat (in vivo); In vitro
Cyclic stretch	Repetitive deformation of the vessel wall due to pulsatile pressure	Increased amplitude and frequency	VSMCs, Adventitial fibroblasts	[22,44,49]	Mouse (in vivo); Rat (in vivo); In vitro
Hydrostatic pressure	Luminal pressure acting perpendicular to the vessel wall	Sustained elevation	All layers	[45,46,47]	Mouse (in vivo); Rat (in vivo); In vitro
Interstitial fluid pressure	Pressure within the tissue interstitium	Elevated (especially in the kidney)	Tissue-resident cells	[48]	Mouse (in vivo)

Abbreviation: VSMCs, vascular smooth muscle cells.

**Table 2:** Cell-type-specific mechanosensor expression and pro-inflammatory functional outcomes in immune subsets under hypertensive mechanical stress.

Immune Cell Subset	Key Mechanosensors	Downstream Signals	Functional Outcomes in Hypertension	Key References	Study Sources
T cells	Piezo1	Ca^2+^ → calcineurin-NFAT	Th17 skewing, IFN-γ/IL-17 production	[58,59,68,69]	Human (in vitro); Mouse (in vivo, in vitro)
Macrophages	TRPV4, Piezo1	Ca^2+^ → NLRP3-IL-1β; NF-κB	M1 polarization, IL-1β release	[6,61,63]	Mouse (in vivo, in vitro)
Dendritic cells	Piezo1	Ca^2+^ → NF-AT/AP-1	Enhanced maturation, IL-6 secretion, Th17 potentiation	[64,70]	Mouse (in vivo, in vitro)
Neutrophils	Piezo1, TRPV4	Ca^2+^ → calpain, NETosis	Endothelial injury via NETs	[3,56,66]	Human (in vitro); Mouse (in vivo, in vitro)
B cells	Piezo1 (expression)	Unknown	Largely unexplored	[71]	Human (in vitro); Mouse (in vivo, in vitro)

Abbreviations: Piezo1, piezo-type mechanosensitive ion channel component 1; TRPV4, transient receptor potential vanilloid 4; NFAT, nuclear factor of activated T cells; NF-κB, nuclear factor kappa-light-chain-enhancer of activated B cells; NLRP3, NLR family pyrin domain containing 3; IL, interleukin; IFN-γ, interferon-gamma; TCR, T cell receptor; NETs, neutrophil extracellular traps; AP-1, activator protein-1.

**Table 3:** Molecular nodes of the mechano-immune activation loop: functional categorization and their hierarchical roles in sustaining positive feedback.

Node	Molecules	Function	Position in the Loop	Key References	Study Sources
Mechanosensing node	Piezo1, TRPV4	Convert physical forces into biochemical signals	Entry (input)	[12,14,19,22]	Human (in vivo, in vitro); Mouse (in vivo, in vitro); Rat (in vivo)
Ca^2+^ node	Calcineurin, CaMK	Second messenger amplification and branching	Transduction	[3,12,96]	Mouse (in vivo); Rat (in vivo); In vitro
Transcriptional node	NFAT, NF-κB, YAP	Drive pro-inflammatory gene expression	Effector initiation	[23,25,26,39]	Mouse (in vivo); Rat (in vivo); In vitro
Inflammatory effector node	IL-17A, IL-1β, IFN-γ	Mediate vascular injury and dysfunction	Effector output	[76,85,97]	Human (in vivo, in vitro); Mouse (in vivo); Rat (in vivo)
Vascular remodeling node	MMPs, TGF-β, Collagen	Alter vascular mechanical properties	Feedback (output to input)	[22,47,49]	Human (in vivo); Mouse (in vivo); Rat (in vivo)

Abbreviations: Piezo1, piezo-type mechanosensitive ion channel component 1; TRPV4, transient receptor potential vanilloid 4; CaMK, Ca^2+^/calmodulin-dependent protein kinase; NFAT, nuclear factor of activated T cells; NF-κB, nuclear factor kappa-light-chain-enhancer of activated B cells; YAP/TAZ, Yes-associated protein/transcriptional coactivator with PDZ-binding motif; AP-1, activator protein-1; IL, interleukin; TNF-α, tumor necrosis factor-alpha; MMPs, matrix metalloproteinases; TGF-β, transforming growth factor-beta; ROS, reactive oxygen species; VSMC, vascular smooth muscle cell.

**Table 4:** Organ-specific mechano-immune microenvironments in hypertension: linking local mechanical cues, sensor expression, and pathological remodeling.

Organ	Primary Mechanical Stress	Affected Cell Types	Key Mechanosensor(s)	Pathological Outcomes	Key References	Study Sources
Kidney	High hydrostatic pressure, tubular shear	Podocytes, mesangial cells, tubular epithelium, resident macrophages	Piezo1, integrins, primary cilia	Glomerulosclerosis, albuminuria, tubulointerstitial inflammation	[45,46,85]	Mouse (in vivo); Rat (in vivo); In vitro
Large arteries	High shear, cyclic stretch	Endothelium, VSMCs, adventitial fibroblasts	Piezo1, TRPV4, integrins	Endothelial activation, VSMC phenotype switching, stiffening	[22,41,49]	Human (in vivo); Mouse (in vivo); Rat (in vivo); In vitro
Heart	Pressure overload, wall stress	Cardiomyocytes, fibroblasts, endothelial cells	Piezo1, TRPV4	Hypertrophy, fibrosis, diastolic dysfunction	[89,113]	Rat (in vivo); Mouse (in vivo); In vitro
Brain	Pulsatile pressure, microvascular shear	Endothelial cells, microglia	Piezo1, TRPV4	BBB disruption, microglial activation, neuroinflammation	[117,119,120]	Mouse (in vivo); In vitro

Abbreviations: Piezo1, piezo-type mechanosensitive ion channel component 1; TRPV4, transient receptor potential vanilloid 4; VSMCs, vascular smooth muscle cells; BBB, blood–brain barrier.

**Table 5:** Preclinical pharmacological tools targeting mechanosensitive channels in the loop: stage of development and experimental model systems.

Target	Tool/Compound	Development Stage	Key References	Study Sources
Piezo1	GsMTx4 (peptide inhibitor), Yoda1 (agonist for research)	Preclinical	[12,45,121]	Mouse (in vivo); Rat (in vivo); In vitro
TRPV4	GSK2193874, HC-067047	Preclinical/Early clinical	[13,16]	Mouse (in vivo); Rat (in vivo); Human (in vivo); In vitro
TRPC6	BI-749327	Preclinical	[18]	Mouse (in vivo); Rat (in vivo); In vitro

Abbreviations: Piezo1, piezo-type mechanosensitive ion channel component 1; TRPV4, transient receptor potential vanilloid 4; TRPC6, transient receptor potential canonical 6; GsMTx4, grammostola spatulata mechanotoxin 4.

**Table 6:** Therapeutic strategies for interrupting the mechano-immune loop: rationale, potential benefits, and translational hurdles.

Strategy	Target/Intervention	Examples	Potential Benefits	Major Challenges	Key References	Study Sources
Direct channel blockade	Piezo1, TRPV4, TRPC6	GsMTx4, GSK2193874, BI-749327	Reduce mechanical entry and downstream inflammation	Off-target effects, poor bioavailability, cell-type non-selectivity	[16,18,45]	Mouse (in vivo); Rat (in vivo); In vitro
Repurposing antihypertensives	ACEIs/ARBs, CCBs, MR antagonists	Lisinopril, amlodipine, spironolactone	Lower BP + anti-inflammatory actions	Indirect, non-specific, may not fully interrupt loop	[125,126]	Human (in vivo); Mouse (in vivo); Rat (in vivo)
Modifying mechanical environment	Strict BP control, arterial compliance improvement	Target SBP <130 mmHg, relaxin, AGE inhibitors	Directly reduce input to the loop	Structural irreversibility, late intervention	[129,130,131]	Human (in vivo); Mouse (in vivo); Rat (in vivo)
Combination or multi-target	Dual inhibition (e.g., Piezo1 + TLR4)	Not yet tested	Potentially synergistic	Redundancy, increased side effects, unknown efficacy	-	Hypothetical

Abbreviations: Piezo1, piezo-type mechanosensitive ion channel component 1; TRPV4, transient receptor potential vanilloid 4; TRPC6, transient receptor potential canonical 6; ACEIs, angiotensin-converting enzyme inhibitors; ARBs, angiotensin II receptor blockers; CCBs, calcium channel blockers; MR, mineralocorticoid receptor; BP, blood pressure; SBP, systolic blood pressure; AGE, advanced glycation end-products; TLR4, toll-like receptor 4.

## Data Availability

No new data were created or analyzed in this study. Data sharing is not applicable to this article.

## Abbreviations

The following abbreviations are used in this manuscript:

ACEIs	angiotensin-converting enzyme inhibitors
Ang II	angiotensin II
ANP	atrial natriuretic peptide
AP-1	activator protein-1
ARBs	angiotensin II receptor blockers
BBB	blood–brain barrier
BKCa	large-conductance calcium-activated potassium channel
BNP	B-type natriuretic peptide
CaMK	Ca^2+^/calmodulin-dependent protein kinase
CCBs	calcium channel blockers
CD40	cluster of differentiation 40
CREB	cAMP response element-binding protein
CRP	C-reactive protein
cPLA2	cytosolic phospholipase A2
CyP-A	cyclophilin A
CysC	cystatin C
DAMPs	damage-associated molecular patterns
DC	dendritic cell
DOCA	deoxycorticosterone acetate
ECM	extracellular matrix
ENaC	epithelial sodium channel
ERK	extracellular signal-regulated kinase
FAK	focal adhesion kinase
GsMTx4	Grammostola spatulata mechanotoxin 4
Hsp60	heat shock protein 60
ICAM-1	intercellular adhesion molecule-1
IFN-γ	interferon-gamma
IKCa	intermediate-conductance calcium-activated potassium channel
IKK	IκB kinase
IL	interleukin
JAK	janus kinase
JNK	c-Jun N-terminal kinase
KCa	calcium-dependent potassium channels
LATS1/2	large tumor suppressor kinase 1/2
LINC	linker of nucleoskeleton and cytoskeleton
MAPK	mitogen-activated protein kinase
MCP-1	monocyte chemoattractant protein-1
MEK	mitogen-activated protein kinase
MHC	major histocompatibility complex
MKK	MAP kinase kinase
MMPs	matrix metalloproteinases
MR	mineralocorticoid receptor
NADPH	nicotinamide adenine dinucleotide phosphate hydrogen
NETosis	neutrophil extracellular trap formation
NFAT	nuclear factor of activated T cells
NF-κB	nuclear factor kappa-light-chain-enhancer of activated B cells
NLRP3	NLR family pyrin domain containing 3
NO	nitric oxide
Nox	NADPH oxidase
NT-proBNP	N-terminal pro-B-type natriuretic peptide
P2X7	P2X purinoceptor 7
PAI-1	plasminogen activator inhibitor-1
Piezo1	piezo-type mechanosensitive ion channel component 1
Piezo2	piezo-type mechanosensitive ion channel component 2
PKC	protein kinase C
PP2A	protein phosphatase 2A
PWV	pulse wave velocity
RAAS	renin–angiotensin–aldosterone system
Rac1	Ras-related C3 botulinum toxin substrate 1
RhoA	Ras homolog family member A
ROS	reactive oxygen species
SGK1	serum/glucocorticoid regulated kinase 1
SKCa	small-conductance calcium-activated potassium channel
SNS	sympathetic nervous system
STAT3	signal transducer and activator of transcription 3
TAC	transverse aortic constriction
TAZ	transcriptional coactivator with PDZ-binding motif
TCR	T-cell receptor
TGF-β	transforming growth factor-beta
Th17	T helper 17 cell
TLR	toll-like receptor
TNF-α	tumor necrosis factor-alpha
Treg	regulatory T cell
TRP	transient receptor potential
TRPC6	transient receptor potential canonical 6
TRPV4	transient receptor potential vanilloid 4
VCAM-1	vascular cell adhesion molecule-1
VSMC	vascular smooth muscle cell
VSMCs	vascular smooth muscle cell layer
YAP	yes-associated protein
Yoda1	piezo1 agonist (research tool)
ZAP70	zeta-chain-associated protein kinase 70
